# Supplementary material for: Personalised therapeutic management of epileptic patients guided by pathway-driven breath metabolomics
Source: Commun Med (Lond). 2021 Aug 2;1:21. doi: 10.1038/s43856-021-00021-3 (PMC9053280; doi:10.1038/s43856-021-00021-3)
Supplement: Supplementary file 1 — Supplementary Materials [file 43856_2021_21_MOESM1_ESM.pdf]

Personalised therapeutic management of epileptic patients guided by pathway-driven breath metabolomics

Authors: Kapil Dev Singh[1,2], Martin Osswald[3], Victoria C. Ziesenitz[1], Mo Awchi[1,2], Jakob Usemann[1], Lukas L. Imbach[3], Malcolm Kohler[3], Diego García-Gómez[4], Johannes van den Anker[1], Urs Frey[1,2], Alexandre N. Datta[1,*], Pablo Sinues[1,2,*]

Affiliations:

[1]University Children's Hospital Basel, University of Basel, Basel, Switzerland.

[2]Department of Biomedical Engineering, University of Basel, Basel, Switzerland.

[3]University Hospital Zurich, University of Zurich, Zurich, Switzerland.

[4]Department of Analytical Chemistry, University of Salamanca, Salamanca, Spain.

[*] Shared corresponding authors: alexandre.datta@ukbb.ch and pablo.sinues@unibas.ch

This supplementary information document contains:

Supplementary Tables 1-4

Supplementary Figures 1-17

Clinical protocol (ID 2017-01537)

Supplementary Table 1: Definitions used to classify clinical outcome of paediatric measurements

	Category		
Class	Side effects	Drug response	Electroencephalography (EEG)
I	No side effects (n = 86)	No seizures or least possible number of seizures for the type of epilepsy (n = 83)	Normal or improved EEG (n = 79)
II	Mild side effects (n = 34)	Reduction in the number of seizures by more than or at least 50%[1] (n = 37)	Reduction of epileptic activity in the EEG by more than or at least 50%[2] (n = 33)
III	Several mild or at least one severe side effect (n = 3)	Reduction in the number of seizures by less than 50%[1] (n = 3)	Reduction of epileptic activity in the EEG by less than 50%[2] (n = 11)

[1]: compared to the phase of their highest seizure activity
[2]: compared to the phase of their highest epileptic activity in the EEG
n denotes the number of measurements

Supplementary Table 2: Different side effects used to classify measurements

Subscales	Items
Cognitive	Slow thinking<br>Memory problems<br>Confusion<br>Poor school results<br>Decreased concentration<br>Attention difficulties
Motor	Unstable walking<br>Poor coordination, clumsiness<br>Falling (not seizure)<br>Speech difficulties
Behavioral	Aggression<br>Hyperactivity<br>Personality change
General neurological	Drowsiness, sleepiness<br>Fatigue, tiredness<br>Dizziness, lightheadedness<br>Headaches
Weight	Increase in appetite<br>Weight gain

Supplementary Table 3: Information about blood analysis methods and samples of ASMs from clinical laboratory of USB

	Blood analysis				Sample	
Drugs	Method	Reference range	Unit	Response time	Material	Minimum quantity (mL) (adults / children)
Ethosuximide (ESX)	LC-MS/MS	283.0 - 708.0	μmol/L	Up to 5 working days	Serum	2.7 / 2.7
Lamotrigine (LTG)	LC-MS/MS	1.3 - 20.5	mg/L		Serum	2.0 / 1.0
Levetiracetam (LEV)	LC-MS/MS	70.0 - 270.0	μmol/L		Serum	2.0 / 1.0
Oxcarbazepine (OXC) and metabolites	LC-MS/MS	3.0 - 35.0*	mg/L	Up to 4 working days	Serum	2.7 / 2.7
Perampanel (PER)	LC-MS/MS	180.0 - 980.0	μg/L	2-3 working days	Serum	2.7 / 2.7
Phenobarbital (PB)	KIMS	15.0 - 40.0	mg/L	3 hours	Serum	1.0 / 0.5
Sultiame (STM)	LC-MS/MS	07.0 - 28.0	μmol/L	2-3 working days	Serum	2.7 / 1.0
Topiramate (TPM)	LC-MS/MS	14.8 - 59.0	μmol/L	2-3 working days	Serum	2.7 / 2.7
Valproic acid (VPA) | Total	EMIT	50.0 - 100.0	mg/L	3 hours	Serum	1.0 / 0.5
Valproic acid (VPA) | Free	GC-MS	5.0 - 10.0	mg/L	2-3 working days	Serum	4.7 / 4.7
Vigabatrin (VIG)	LC-MS/MS	2.0 - 10.0	mg/L		Serum	0.5 / 0.5

* for 10-hydroxycarbazepine
KIMS = Kinetic interaction of microparticles in solution immunoassay
EMIT = Enzyme-multiplied immunoassay

Supplementary Table 4: Information about 11 features used for predicting serum concentrations of VPA

	Assigned			Identified molecule	
Measured m/z	Formula	Adduct	Error (ppm)	Formula (M)	Name
115.11176	$C_7H_{15}O$	$[M + H]^+$	0.159		
116.11508	$C_6{}^{13}CH_{15}O$	$[M + 1 + H]^+$	−0.142	$C_7H_{14}O$	3-Heptanone*
132.13834	$C_7H_{18}ON$	$[M + NH_4]^+$	0.373		
129.09102	$C_7H_{13}O_2$	$[M + H]^+$	0.107	$C_7H_{12}O_2$	Heptanedione#
130.09442	$C_6{}^{13}CH_{13}O_2$	$[M + 1 + H]^+$	0.454		
143.10659	$C_8H_{15}O_2$	$[M + H]^+$	−0.463		
144.10991	$C_7{}^{13}CH_{15}O_2$	$[M + 1 + H]^+$	−0.701		
145.11081	$C_8H_{15}O^{18}O$	$[M + 2 + H]^+$	−0.634	$C_8H_{14}O_2$	4-OH-γ-Lactone*
160.13321	$C_8H_{18}O_2N$	$[M + NH_4]^+$	0.029		
161.13655	$C_7{}^{13}CH_{18}O_2N$	$[M + 1 + NH_4]^+$	−0.063		
144.11106	$C_7{}^{13}CH_{15}O_2$	$[M + 1 - H]^-$	−0.332	$C_8H_{16}O_2$	VPA

* denotes molecules confirmed by LC-MS/MS
# denotes hypothesized molecule (2,3- or 2,5-heptanedione)

Supplementary Fig. 1: Distribution of breath measurement numbers

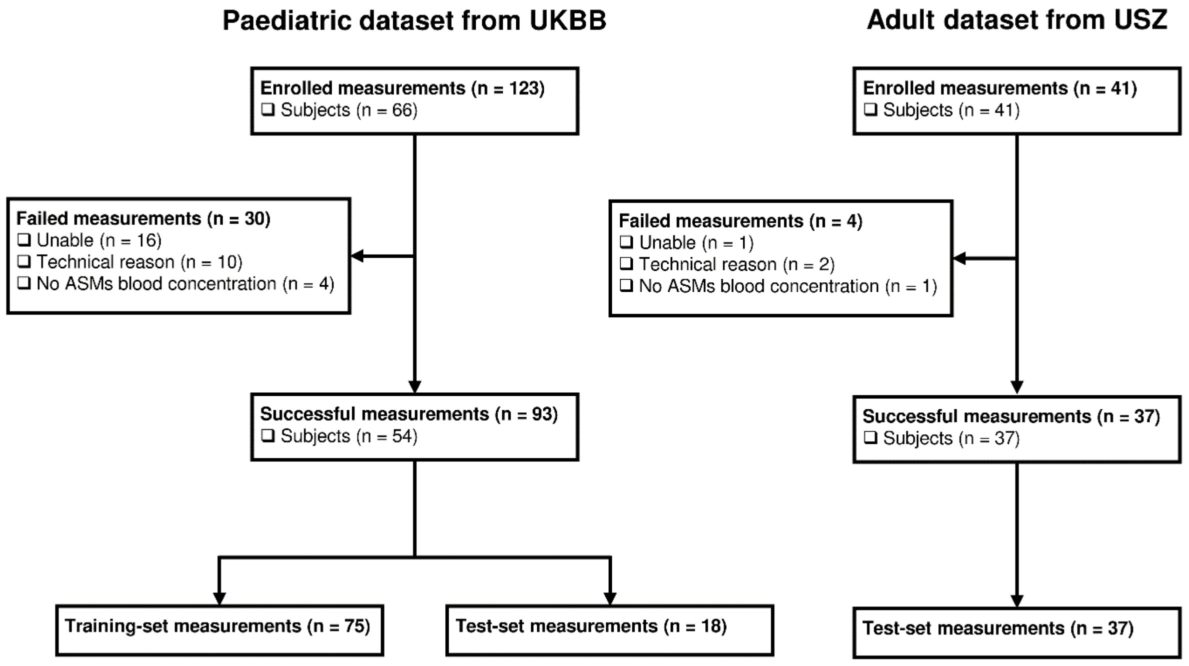

Figure shows the number of breath measurements enrolled and finally used in this study for paediatric and adult datsets.

Supplementary Fig. 2: Pictorial representation of a child exhaling into SESI-HRMS system

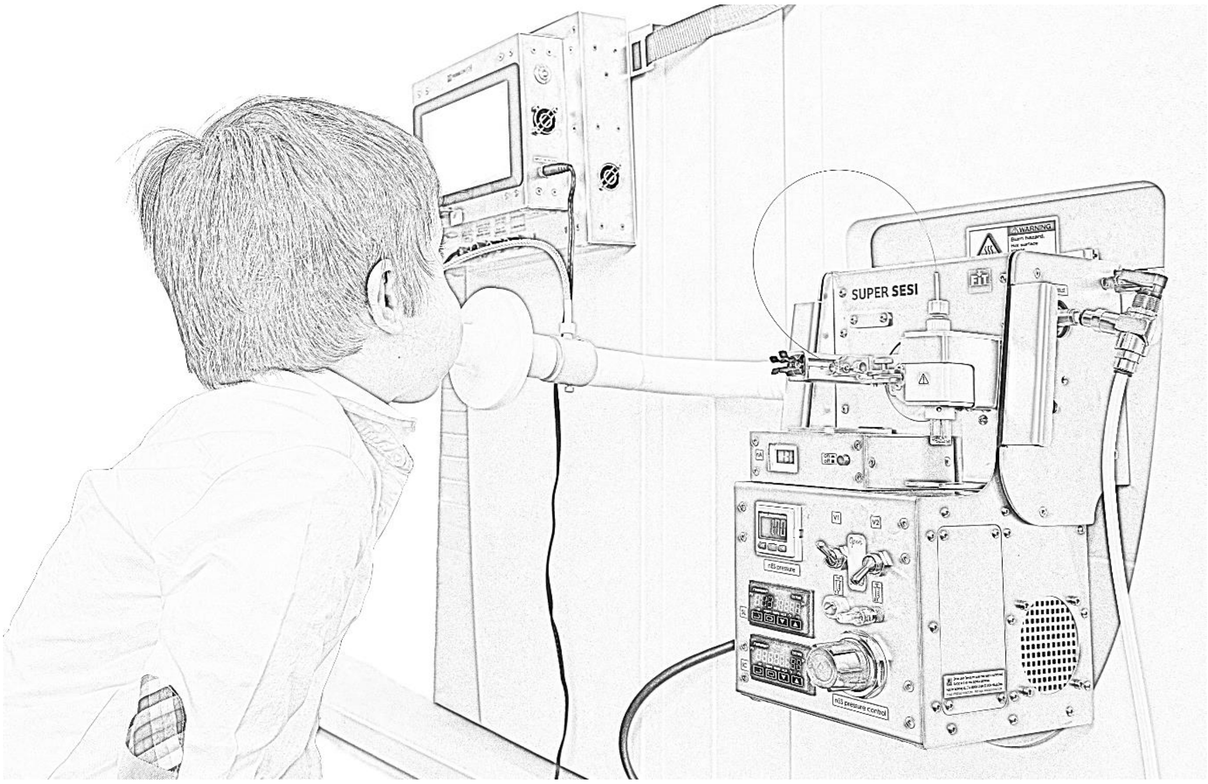

Supplementary Fig. 3: Sample size calculation

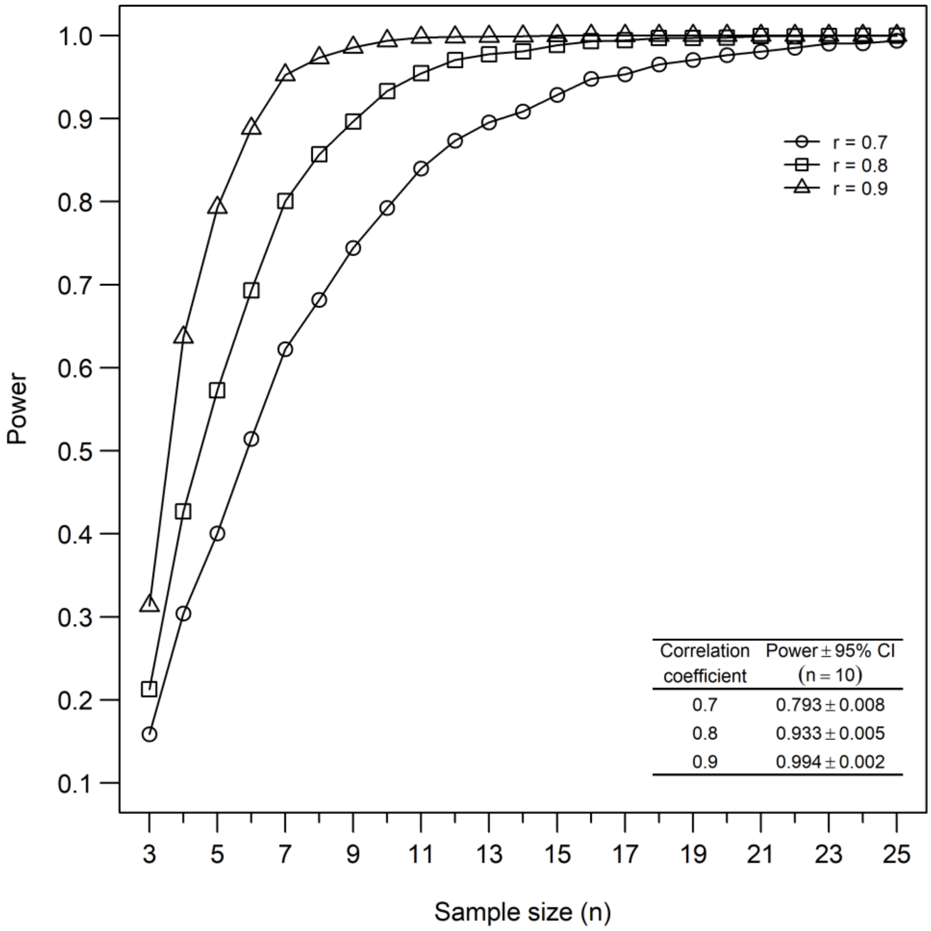

Figure shows the relationship between power and sample size (n) assessed based on Monte Carlo simulations (10000 times) for correlation cofficients (r) between two variables with samples sizes ranging between 3 and 25. This was done prior to the start of this study and three possible correlation coefficients (i.e. 0.7, 0.8, and 0.9) were considered based on previous studies (suggesting a correlation in range of 0.7 to 0.9 is possible between measurements in breath and blood). Table in the lower right shows the possible power at various correlation coefficient. Based on this we estimated that with 10 measurements our predictions will have power between 0.79 - 0.99, depending on the correlation in breath and blood levels.

Supplementary Fig. 4: ComBat was used to correct for known batch and site effects

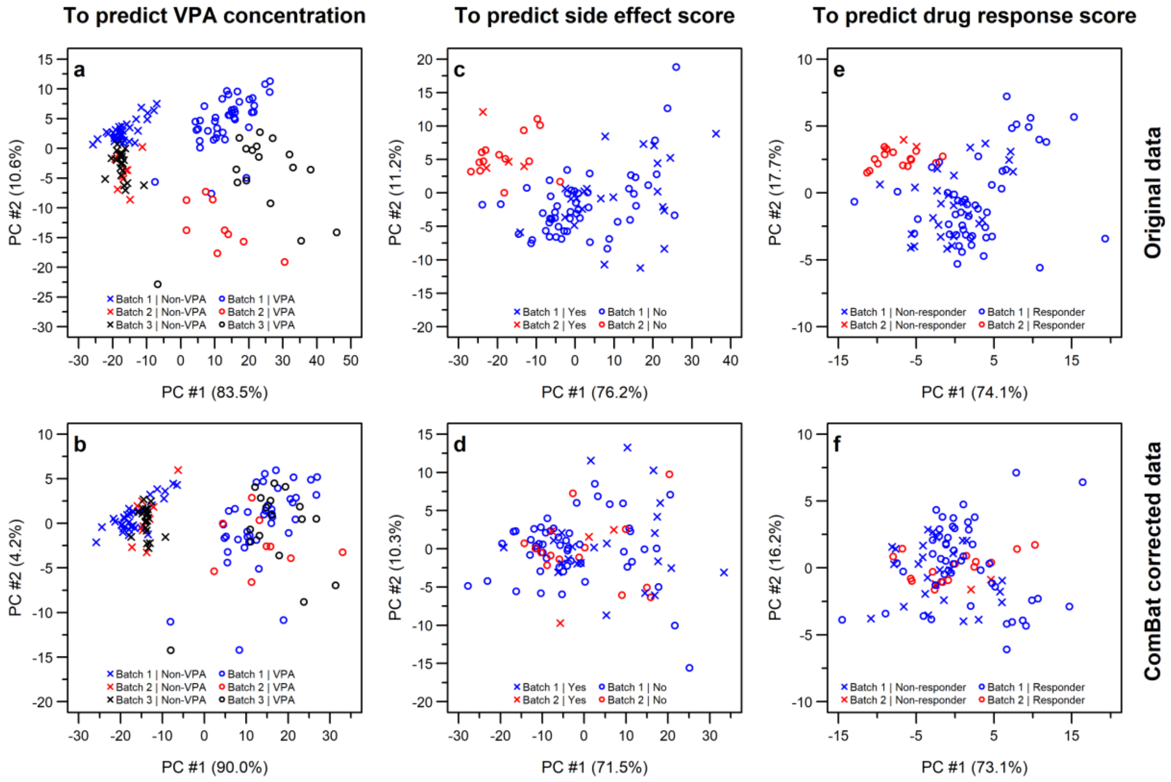

Figure shows the scatter plot between first and second principle component from the original and ComBat corrected final data matrices used to predict VPA concentration (**a, b**), side effect scores (**c, d**), and drug response scores (**e, f**). Batch 1 is UKBB training-set, Batch 2 is UKBB test-set (a different set of mouth-piece was used), and Batch 3 is USZ test-set (different site). ComBat from R-package sva (version 3.34.0) was used with default arguments, but UKBB training-set (i.e. Batch 1) was used as reference batch.

Supplementary Fig. 5: Workflow to determine the regression model with best performance

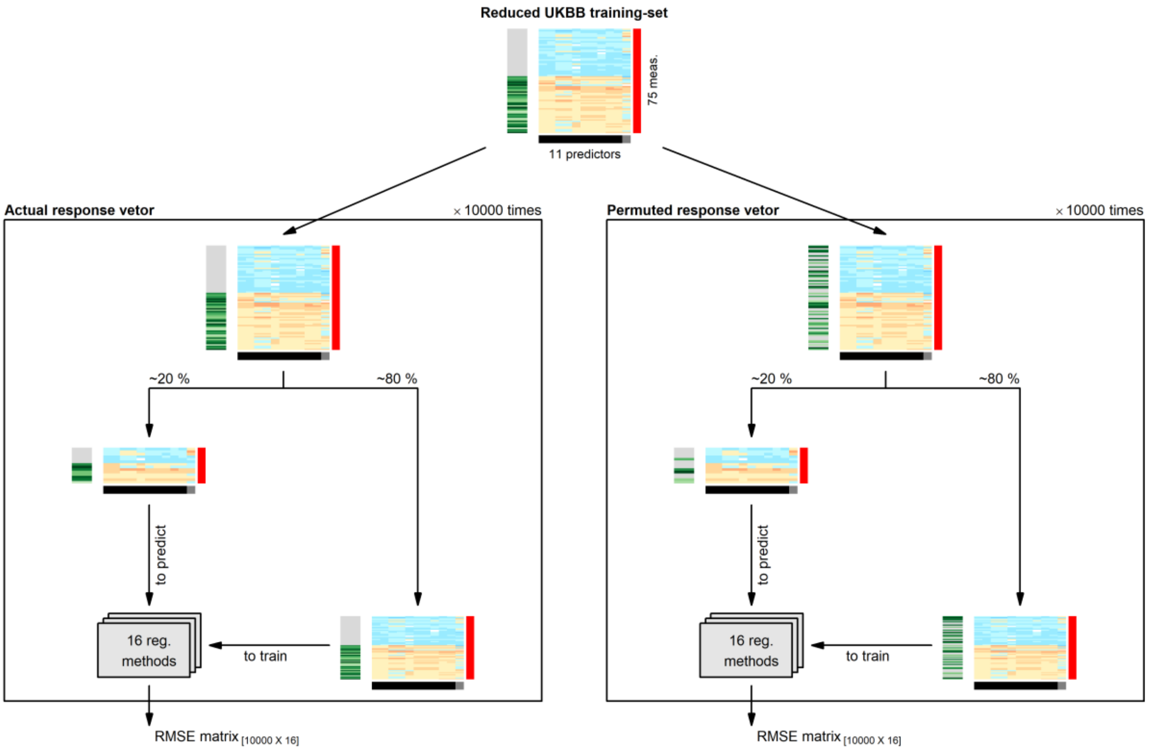

The reduced UKBB training-set (i.e. training-set with only 11 features) with actual and permuted response vectors was partitioned into 20% and 80% of measurements. The data with 80% of measurements was then used to train 16 different regression models (see Supplementary Fig. 6) and then those models were used to predict response in the remaining 20% of measurements. This whole procedure was repeated 10,000 times. Data generate from this procedure was used to determine best performing regression model (see Supplementary Fig. 6).

Supplementary Fig. 6: Determination of best performing regression algorithm

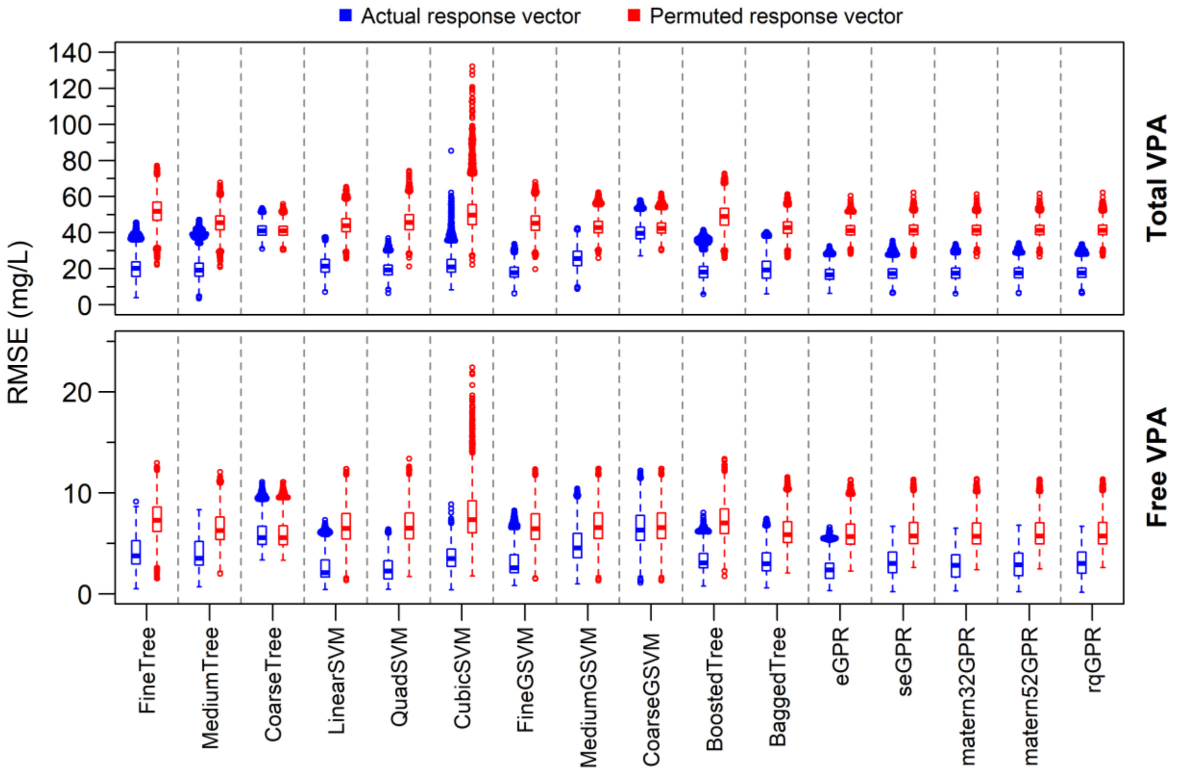

Data shown are tukey style box plots of root mean square error (RMSE) for prediction of actual and permuted response vector based on 11 VPA related features using just reduced training-set from 16 different regression models available in MATLAB 2019b. Since GPR based models in general perform better than others, we used eGPR as our final prediction model.

Supplementary Fig. 7: Exhaled breath metabolites tend to be upregulated in patients with side effects and appears to be downregulated in non-responders

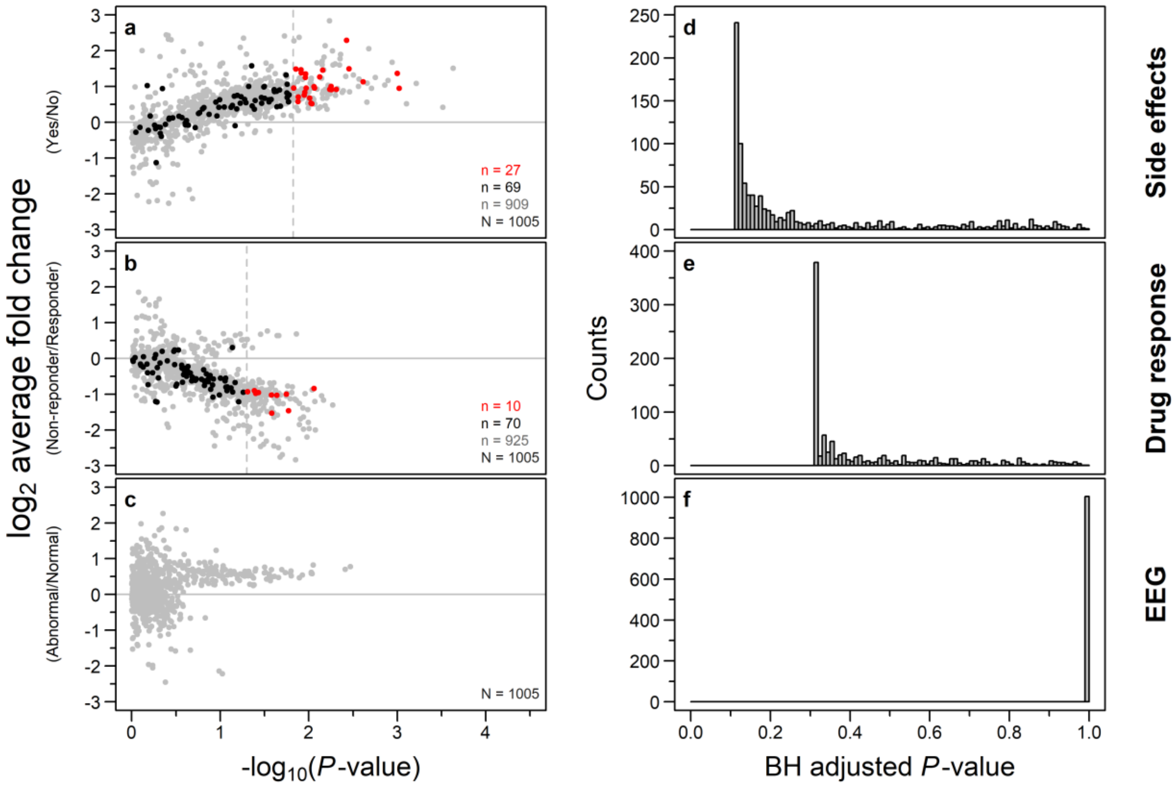

a-c Shows the volcano plots after two sample t-test between training-set measurements of side effects vs no side effects (**a**), non-responders vs responders (**b**), and abnormal vs normal EEG (**c**). Each data point (N = 1005) is an ion measured in SESI-HRMS. Horizontal solid grey line represents the no change and vertical dashed grey line represents *P*-value cut-off of 0.015 in panel **a** and 0.05 in panel **b**. Different (data specific) *P*-value cut-offs were used to make sure ~10% of total ions appears in significant list, to allow proper downstream analysis via MetaboAnalystR. Coloured points represent ions corresponding to compounds from significantly enriched pathways (top-right quadrant in Fig. 5), where red points are significant and black points are not, actual numbers of these and remaining points are denoted by n at the bottom right side of each panel. **d-f** Distribution of BH-adjusted *P*-values of two sample t-test for side effects (**d**), drug response (**e**), and EEG (**f**) based classification of measurements.

Supplementary Fig. 8: Additional information about prediction models for side effects and drug response scores

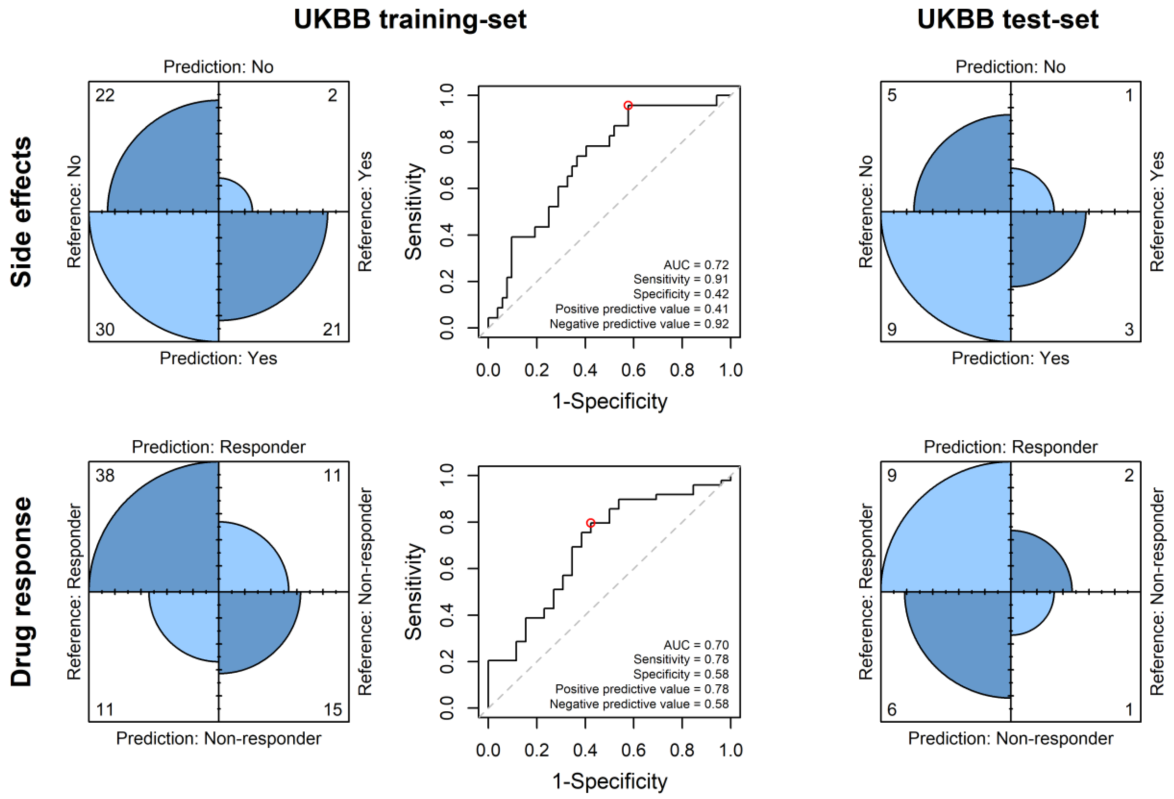

Figure shows the confusion matrices (as Fourfold plot) and receiver operating characteristic (ROC) curves for side effects and drug response from UKBB training-set and confusion matrices for UKBB test-set. Predicted scores were assigned to binary classes using a cut-off based on maxium value of Youden’s index in the UKBB training-set (red open circle in ROC curves).

Supplementary Fig. 9: Additional information about demographics and VPA serum levels in paediatric participants

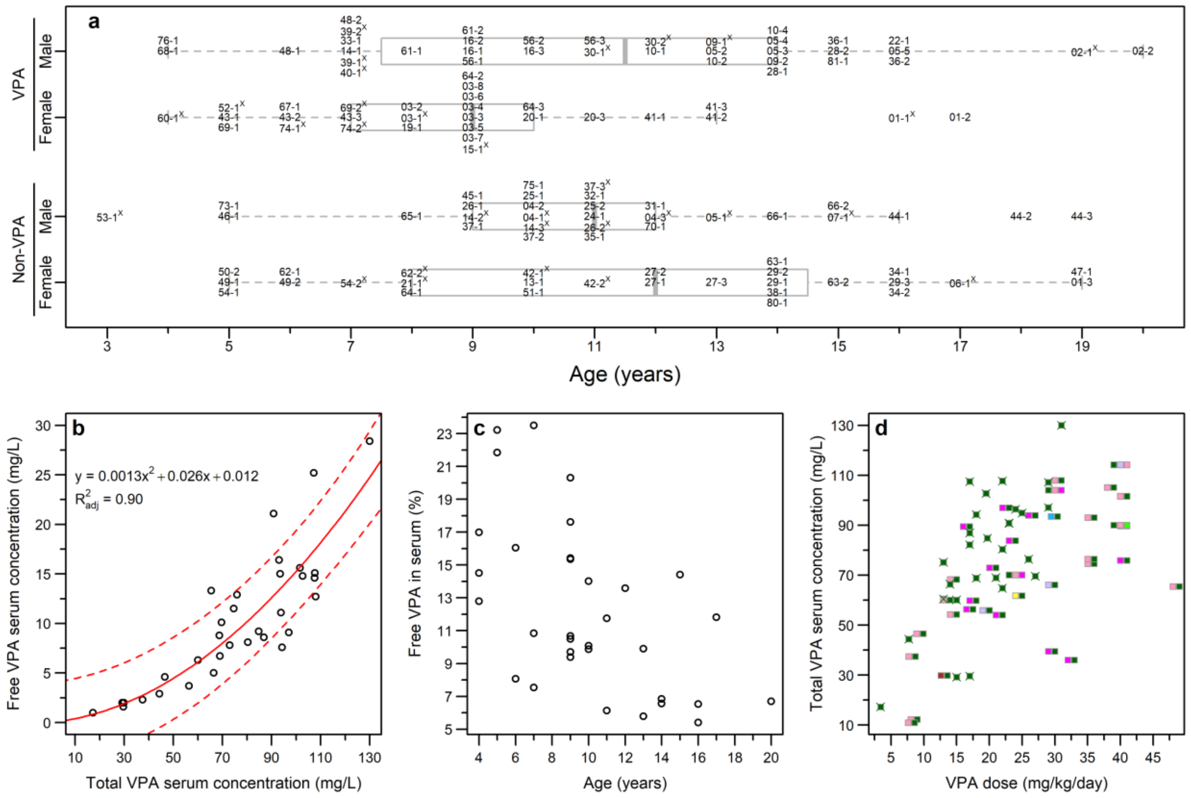

a Distribution of age among paediatric participant. Tukey style boxplots for age of participants are grouped by gender and measurements with VPA and without VPA, boxes are overlaid with actual data points, labelled as subject number-visit number (× denotes measurement with unsuccessful breath test). **b** Shows the widely known non-linear relationship between total and free VPA, solid red line shows the quadratic fit line along with 95% CI as dashed red lines. **c** In paediatric subjects of this study, free VPA shows an inverse relationship with the age, which can be explained by the increasing levels of serum albumin in children[1]. **d** Our data also shows another commonly observed trend that relationship between dose of VPA and serum concentrations of total VPA are highly dependent on other concomitant ASMs (each data point is one measurement where ASM information is represented by using same colour key as in Fig. 2, additionally measurements with mono-therapy VPA are labelled with cross symbol). All of these analyses suggest that this group of patients is a representative real-life sample of unselected hospital outpatients.

Supplementary Fig. 10: Distribution of paediatric measurements among ASMs

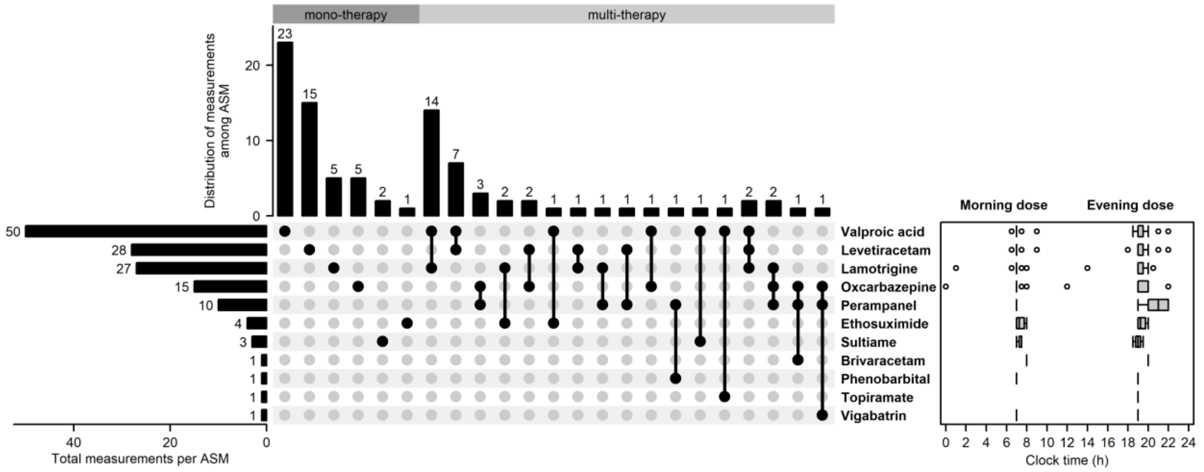

Upset plot[2] representing distribution of successful paediatric measurements among ASMs. Number of measurements shared between different ASMs are shown in the top vertical bar graph and the specific ASMs in each set are indicated with solid points below the bar graph. Total number of measurements for each ASM are shown on the left horizontal bar graph. On the right side a box plot shows the time distribution when subject takes there ASMs. For all mentioned drugs except Topiramate, subjects normally take two dosage a day, approximately 12 hours apart.

Supplementary Fig. 11: High inter and intra-individual heterogeneity in the activity of β and ω_1-oxidation pathways of VPA metabolism

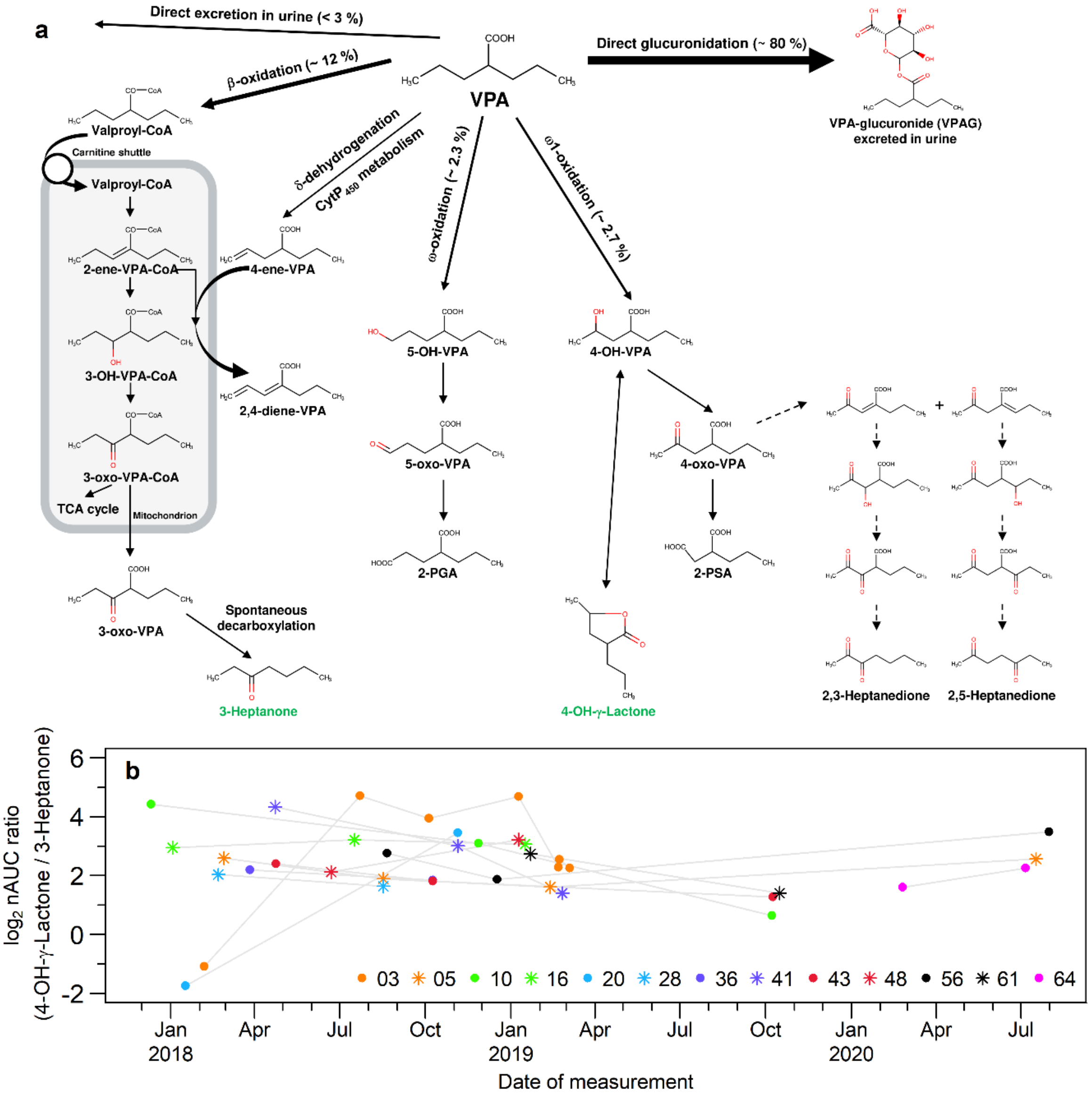

a Schematic representation of VPA metabolism (based on[3–5]), molecules labelled in green belongs to predictors of importance (see Fig. 3 and Supplementary Table 4). Bottom right portion with dashed arrows shows the proposed route for either 2,3- or 2,5-heptanedione formation. We hypothesize this metabolite would be produced downstream the ω1-oxidation route by a subsequent process of oxidation, dehydrogenation, and spontaneous decarboxylation reactions that have been well described for other routes in the metabolism of VPA. **b** Data shows the time profile of β/ω_1-oxidation of VPA assessed based on ratio of 4-OH-γ-lactone and 3-heptanone breath-signal for subjects (taking VPA) with multiple visits. Subject 03 and 20 appears to have significant within-subject variability as compared to other.

Supplementary Fig. 12: Zoomed-in average mass spectra for 11 selected features from training-set breath-MS measurements

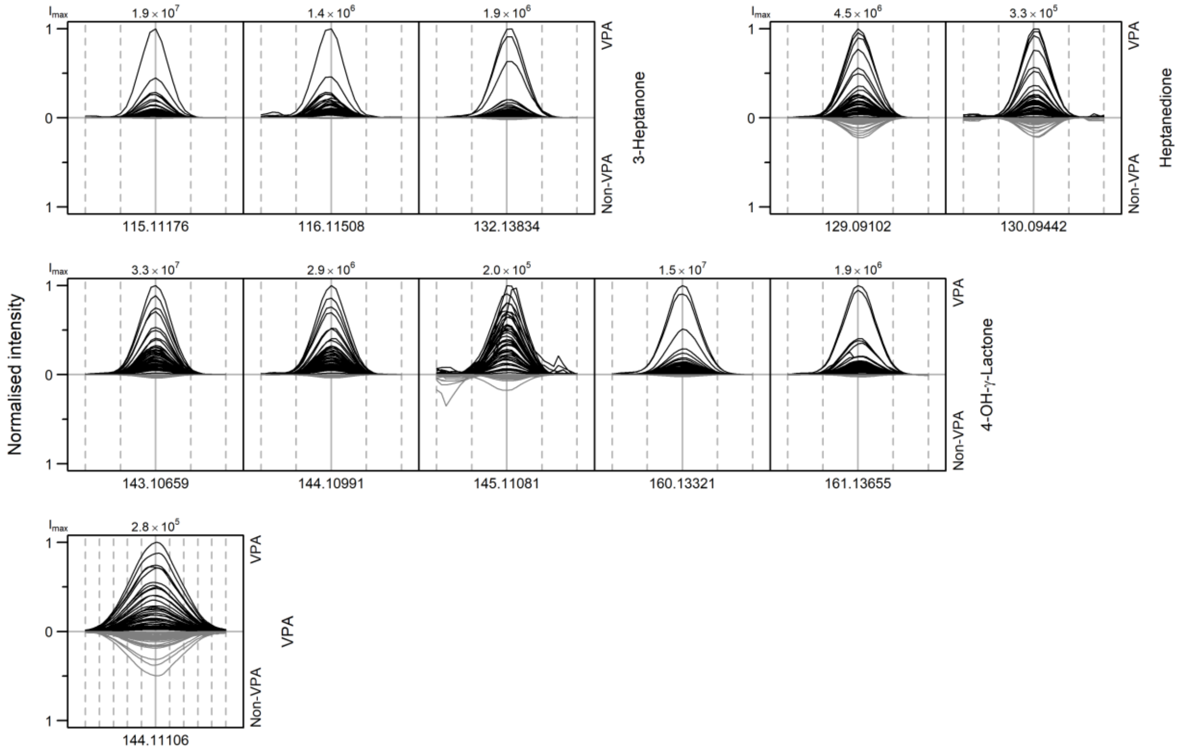

All features are grouped according to their corresponding molecule. Spectra from measurements with VPA are shown in black, whereas spectra from non-VPA measurements are shown in grey (Non-VPA spectra are inverted to ease visual inspection). I_{max} denotes the maximum intensity (AU) value for each feature. The solid vertical grey line represents the expected mass and dashed vertical grey lines represent ± 5 ppm of error. The mass range in x-axis spans over ± 10 ppm for ions in positive mode, however it covers ± 25 ppm for negative mode m/z 144.11106 (due to the use of lower FT resolution in negative mode).

Supplementary Fig. 13: Prediction of total and free VPA serum concentration in non-VPA and VPA takers

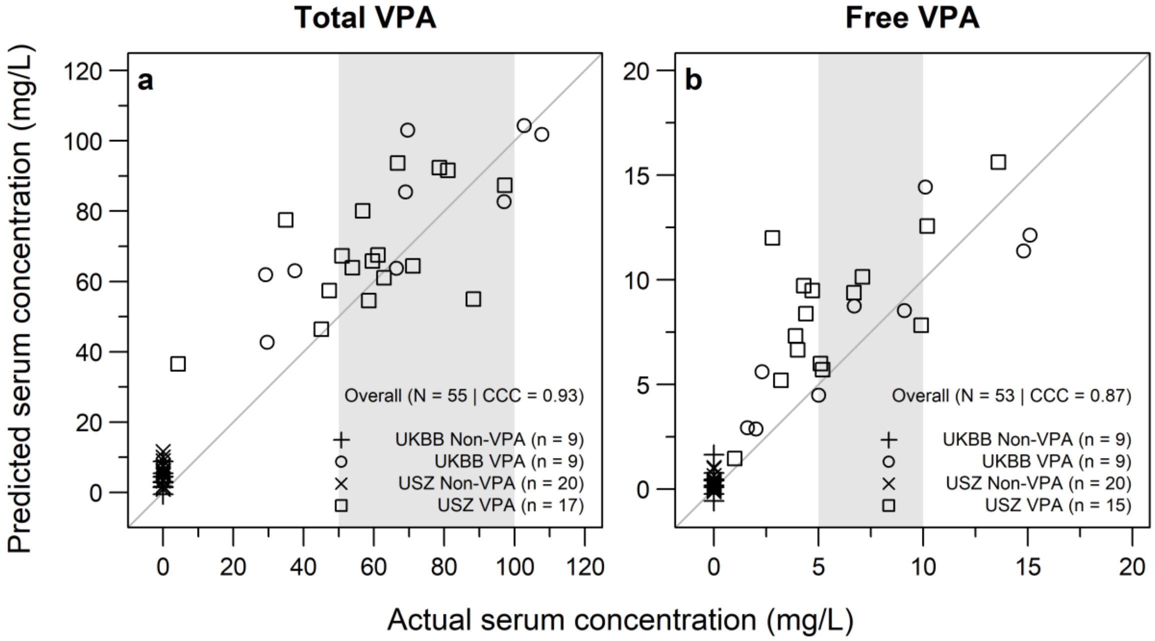

Figure shows the prediction of total (**a**) and free (**b**) VPA serum concentration of independent test-set containing paediatric subjects from UKBB and adult subjects from USZ. Vertical grey box shows the reference therapeutic range of 50-100 mg/L for total VPA and 5-10 mg/L for free VPA. Solid grey line represents the identity (y = x) line.

Supplementary Fig. 14: Number of training-set measurements annotated with side effects, non-responders, and abnormal EEG out of the total measurements

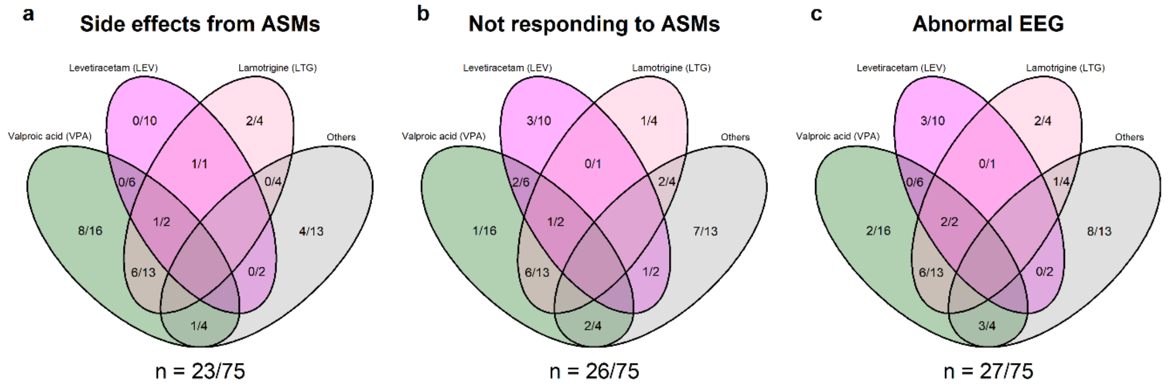

Training-set measurements of patients are grouped into four sets based on ASMs namely: VPA, LEV, LTG, and others. Each region shows the proportion of ASM takers with side effects (**a**), non-responders (**b**), and abnormal EEG (**c**) out of the total measurements. At the bottom of each panel n denotes the proportion of measurements in annotated category out of the total measurements. Note, 5 out of 6 measurements in the intersect of VPA and LTG among different Venn diagrams are same.

Supplementary Fig. 15: Prediction of total and free VPA using different combinations of predictors

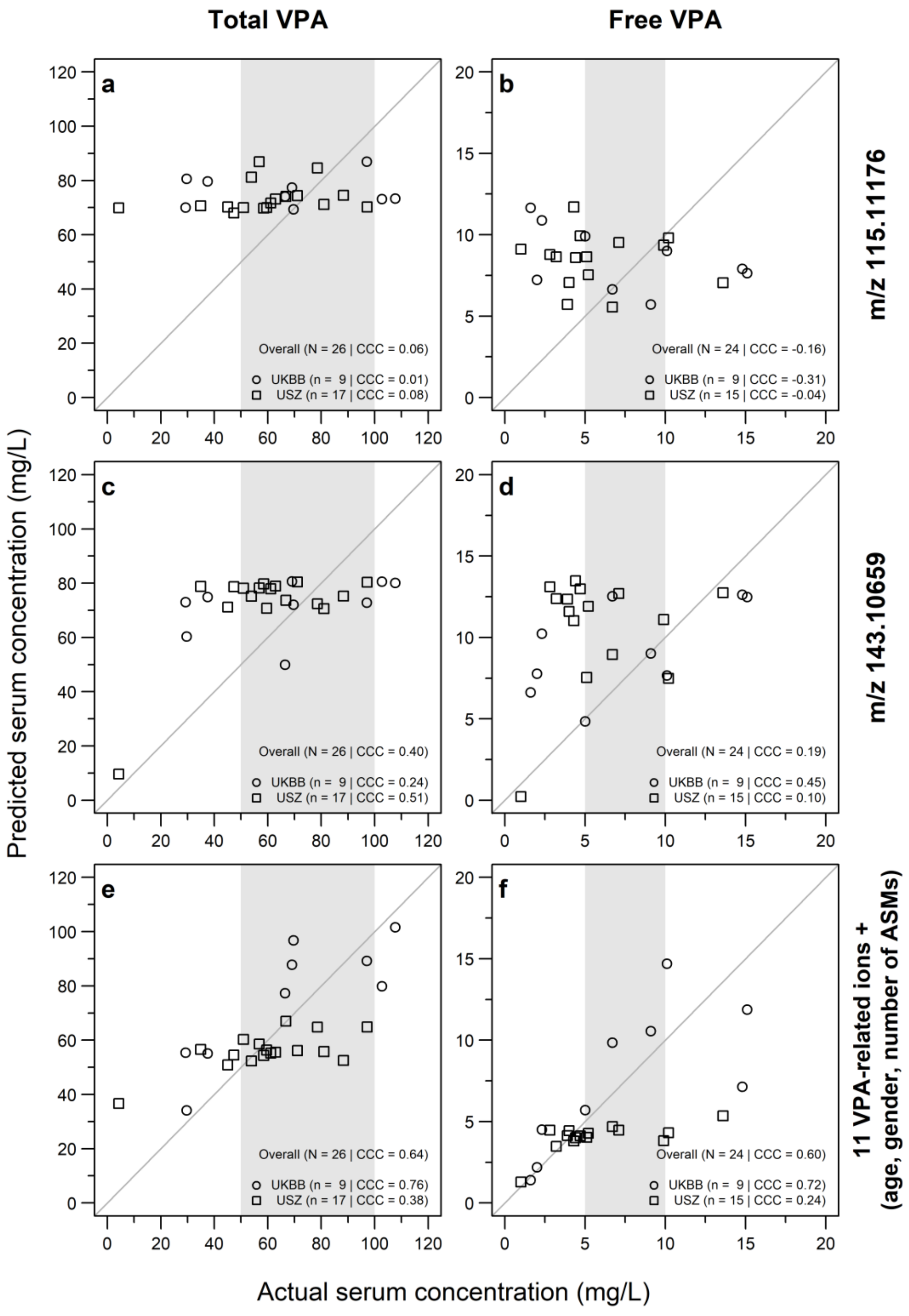

Figure shows the prediction of total (**a**, **c** and **e**) and free (**b**, **d** and **f**) VPA serum concentration of independent test-set. Top row (**a, b**) is based on just m/z 115.1118 as predictor, middle row (**c, d**) is based on only m/z 143.1066 as predictor, and finally bottom row (**e, f**) is based on 11 VPA-related ions plus age, gender and number of ASMs as predictors. Vertical grey box shows the reference therapeutic range of 50-100 mg/L for total VPA and 5-10 mg/L for free VPA. Solid grey line represents the identity (y = x) line.

Supplementary Fig. 16: Distribution of mean nAUC of 11 selected features in paediatric (UKBB site) and adult (USZ site) subjects between VPA and non-VPA takers

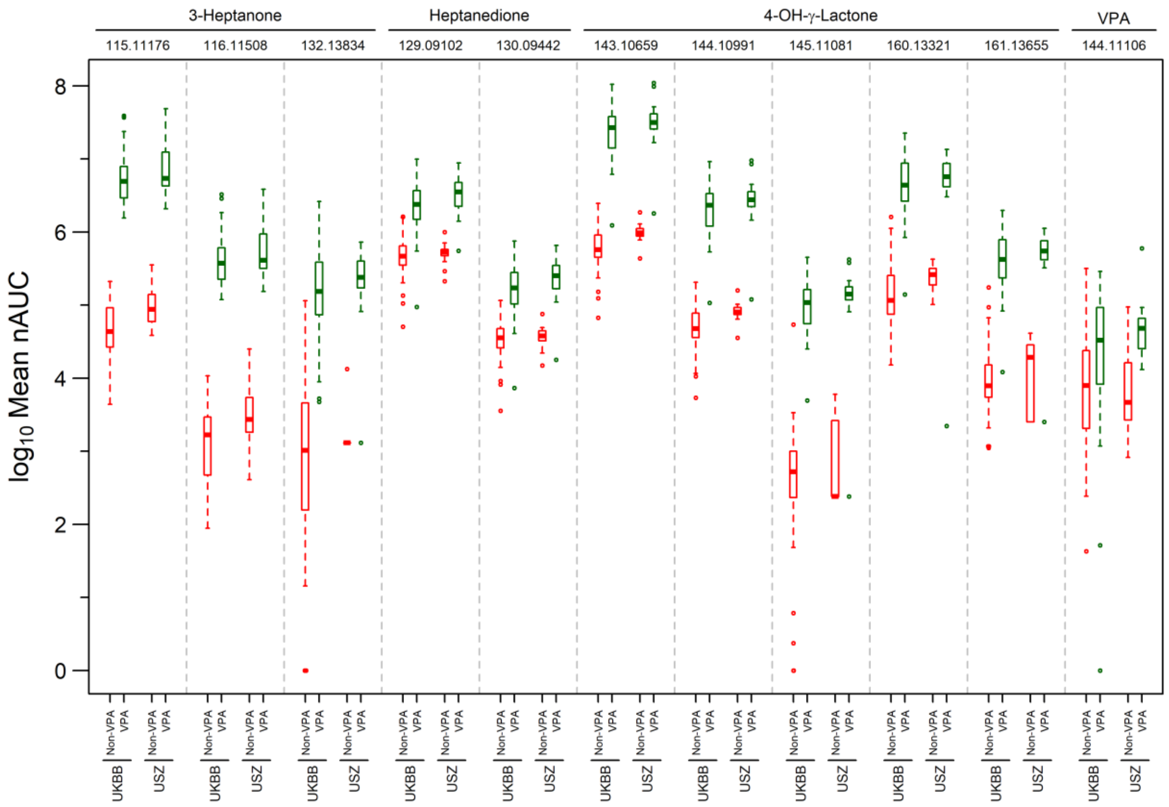

Supplementary Fig. 17: Free VPA blood levels appears to have higher fraction of measurements outside the therapeutic range than total VPA levels

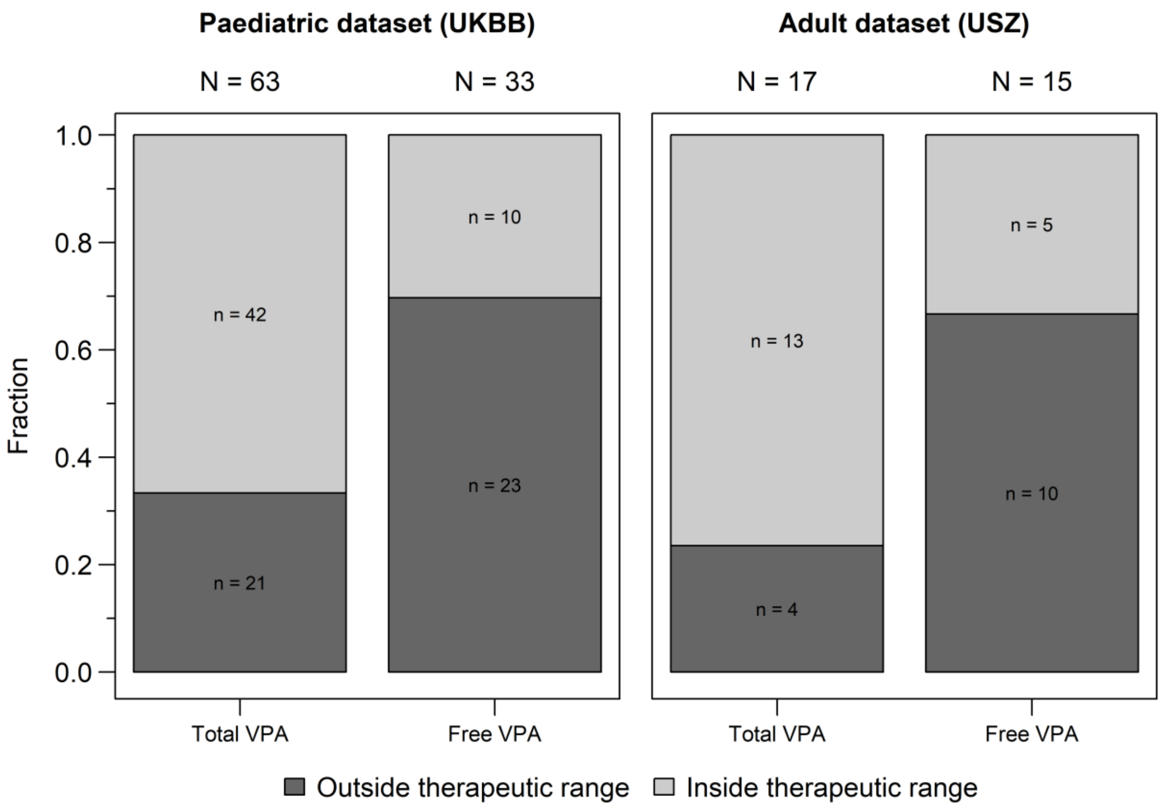

Figure shows the stacked bar plots representing the fraction of measurements outside and inside of the therapeutic range for total (50-100 mg/L) and free (5-10 mg/L) VPA. N denotes the total number of measurements in each bar and n denotes the number of measurements in each fraction. Data includes all measurements from paediatric and adult datasets.

References

1. Weaving, G., Batstone, G. F. & Jones, R. G. Age and sex variation in serum albumin concentration: an observational study. *Ann. Clin. Biochem.* **53**, 106–11 (2016).

2. Lex, A., Gehlenborg, N., Strobelt, H., Vuillemot, R. & Pfister, H. UpSet: Visualization of Intersecting Sets. *IEEE Trans. Vis. Comput. Graph.* **20**, 1983–92 (2014).

3. Ponchaut, S., van Hoof, F. & Veitch, K. In vitro effects of valproate and valproate metabolites on mitochondrial oxidations. Relevance of CoA sequestration to the observed inhibitions. *Biochem. Pharmacol.* **43**, 2435–42 (1992).

4. Erhart, S. *et al.* 3-Heptanone as a potential new marker for valproic acid therapy. *J. Breath Res.* **3**, 016004 (2009).

5. Lheureux, P. E. R., Penaloza, A., Zahir, S. & Gris, M. Science review: carnitine in the treatment of valproic acid-induced toxicity **-** what is the evidence? *Crit. Care* **9**, 431–40 (2005).

Following pages contain the complete clinical protocol (ID 2017-01537) approved by the Ethics Committee of North-western and Central Switzerland.

Research Plan

Feasibility of Therapeutic Drug Monitoring Guided by Exhaled Breath Analysis: A pilot study (Breath-TDM)

Type of Research Project:	Research project in which biological material is sampled from humans and/or health-related personal data is collected.
Risk Categorisation:	Risk Category A
Project Identifier:	Breath-TDM
Project Leader:	Prof. Dr. Pablo Sinues, PhD<br>University Children's Hospital Basel (UKBB)
Health condition / problem	Therapeutic Drug Monitoring of anti-seizure medication and/or chemotherapy and/or antibiotics in pediatric patients
Project Duration	Recruitment start date 01.11.17.<br>Project duration: undefined
Project Plan Version and Date:	V4.3<br>30/10/2019

ACCESS TO RESEARCH DOCUMENTS
Respecting access to these research documents is not in conflict with applicable transparency rules.

TABLE OF CONTENTS

SYNOPSIS (SUMMARY) 6
ABBREVIATIONS 7
SCHEDULE OF ASSESSMENTS 8
1. ADMINISTRATIVE STRUCTURE 9
2. ETHICAL AND REGULATORY ASPECTS 10
2.1 Ethical Conduct of Study 10
2.2 Risk categorisation 10
2.3 Ethics Committee (EC) and Competent Authorities (CA), FOPH 10
2.4 Participant Information and Informed Consent 10
2.5 Participant privacy and safety 11
2.6 Early termination of project 11
2.7 Amendments, Changes 11
3. INTRODUCTION 12
3.1 Background 12
3.2 Rationale for the research project 12
3.3 Risk-Benefit Assessment 12
4. OBJECTIVES, ENPOINTS/OUTCOMES AND OTHER STUDY VARIABLES 12
4.1 Objectives 12
4.2 Primary and secondary endpoint/outcome(s) 12
4.3 Other study variables 13
5. PROJECT DESIGN 14
5.1 Type of research and general project design 14
5.2 Procedures 14
5.2.1 Overview 14
5.2.2 Study description 14
5.3 Recruitment and Screening 16
5.4 Methods of minimising bias 16
6. PROJECT POPULATION 17
6.1 Inclusion criteria 17
6.2 Exclusion criteria 17
6.3 Criteria for withdrawal / discontinuation of participants 17
7. PROJECT ASSESSMENTS 18
7.1 Project flow chart(s) / table of procedures and assessments 18
7.2 Assessments of primary endpoint/outcome 18
7.3 Assessment of secondary endpoints/outcomes 18
7.4 Assessment of other study variables 18
7.5 Assessment of safety and reporting 18
7.5.1 Definition of Serious Events (SEs) 18
7.5.2 Assessment and Documentation of SEs 19
7.5.3 Reporting of SEs, Safety and Protective Measures 19
8. STATISTICAL METHODOLOGY 19
8.1 Determination of Sample Size 19
8.1.1 Summary of the literature 19
8.1.2 Hypothesis 19
8.1.3 Sample size calculation 20
8.1.4 Drop-out considerations 21
8.1.5 Final sample size for the primary endpoint 21
8.2 Data processing 21
8.3 Planned analysis 21
8.3.1 Datasets to be analysed 21
8.3.2 Handling of missing data 21
8.3.3 Ancillary analysis 21
8.3.4 Deviations from the original statistical plan 21
9. DATA AND QUALITY MANAGEMENT 22
9.1 Data handling and record keeping / archiving 22
9.2 Confidentiality, Data Protection 22
9.3 Coding 22
9.4 Archiving and Destruction 22
10. PUBLICATION AND DISSEMINATION POLICY 23
10.1 Publication of results 23

10.2 Data sharing 23
11. FUNDING AND SUPPORT **23**
12. INSURANCE **23**
13. REFERENCES **23**
14. APPENDICES **24**

SYNOPSIS (SUMMARY)

**Project Leader**	Prof. Dr. Pablo Sinues, PhD
**Project Title:**	Feasibility of Therapeutic Drug Monitoring Guided by Exhaled Breath Analysis: A pilot study
**Short Title / Project ID:**	Breath-TDM
**Project Plan Version and Date:**	Version 4.3 (30.10.2019)
**Risk categorisation:**	Risk category A according to ordinance HRO Art.7
**Type of Research:**	Research project in which biological material is sampled and health-related personal data is collected. Coded data will be used.
**Project design:**	Single center, observational prospective pilot study
**Background and Rationale:**	Therapeutic drug monitoring (TDM) is defined as measuring concentrations of a drug at one or more time points in a biological matrix after a dose. The purpose of TDM is to individualize the dose of a specific drug to achieve maximum efficacy and minimal toxicity of its use.
**Objective(s):**	To compare the analysis of drugs and their metabolites in exhaled breath with the current standard of care using plasma samples for TDM of drugs with a narrow therapeutic range in order to assess feasibility for clinical care.
**Endpoint(s):**	Primary endpoint:<br>1) Correlation between drug concentrations in plasma/serum as measured by gold-standard methods and breath concentrations as measured by SESI-MS.<br><br>Secondary endpoints:<br>2) Evaluation of response to medication and drug toxicity as a function of blood/breath drug concentrations.
**Inclusion / Exclusion criteria:**	Inclusion criteria:<br>1. Age 0 to 22 years old, in 3 subgroups (0-5 yrs, 6-12 yrs, 13-22 yrs)<br>2. Treatment with anti-seizure medication(s) and/or chemotherapeutic agents and/or antibiotics that need therapeutic drug monitoring (TDM).<br>3. Blood sampling for TDM is required per standard care.<br><br>Exclusion criteria:<br>1. Patients not capable of providing an exhalation into the mass spectrometer nor their breath can be sampled with off-line methods<br>2. Inability of the care givers to understand the study or provide informed consent
**Project assessments, procedures:**	We will analyze patients' breath using Secondary Electrospray Ionization-Mass Spectrometry (SESI-MS). The method is totally pain-less and non-invasive.
**Number of Participants:**	150 participants
**Project Duration, schedule:**	Recruitment start date 01.11.17.<br>Project duration: undefined and extended until all 150 participants are included
**Project Centre:**	This is a single-center study carried out at University Children's Hospital Basel (UKBB).
**Statistical Considerations:**	We will determine whether there is a significant Pearson's correlation coefficient between blood and breath drug concentrations.
**Other methodological Considerations:**	Not applicable
**Risk-Benefit statement:**	This project poses a minimal risk to the patients as their exhaled air will be analyzed using a simple and non-invasive breath test and in the inclusion criteria we included patients having blood sampling already for clinical purposes. Hence, the risk associated with this study is not higher than the minimal risk for the pediatric population. The SESI source is a non-invasive consumer device and commercially available in Switzerland. The potential benefit is large as it may unlock an alternative non-invasive and potentially more precise method for conducting TDM.

ABBREVIATIONS

DoH	Declaration of Helsinki
EC	Ethics Committee
EGEP	Essentials of Good Epidemiological Practice
FOPH	Federal Office for Public Health
HRA	Federal Act on Research involving Human Beings (Human Research Act, HRA)
HRO	Ordinance on Human Research with the Exception of Clinical Trials (Human Research Ordinance, HRO)
ID	Identification
IIT	Investigator-initiated Trial
SE	Serious event
STROBE	Strengthening the reporting of observational studies in epidemiology
UKBB	Universitäts-Kinderspital beider Basel
USB	Universitätsspital Basel
CRF	Case report form
SESI-MS	Secondary Electrospray Ionization-Mass Spectrometry

SCHEDULE OF ASSESSMENTS

Project Periods			
Visit	1	2	3
Days until/ after Breath test	Day -30 to -1	Day 0 **Breath test**	Day 1-30
Clinician explains study to patient and parents/legal representatives	x		
Eligible patient contacted by study staff	x		
Inclusion/ exclusion criteria assessed by investigator	x		
Patient information and informed consent	x		
Questionnaire		x	x (if not completed on Day 0)
Plasma/blood analysis for TDM		x	

1. ADMINISTRATIVE STRUCTURE

Project Leader and Coordinating researcher

2. ETHICAL AND REGULATORY ASPECTS

2.1 Ethical Conduct of Study

The research project will be carried out in accordance to the research plan and with principles enunciated in the current version of the Declaration of Helsinki (DoH), the Essentials of Good Epidemiological Practice issued by Public Health Schweiz (EGEP), the Swiss Law and Swiss regulatory authority's requirements as applicable. The ethics committee (EC) and regulatory authorities will be informed about project start and termination.

2.2 Risk categorisation

This study is a category A research project involving persons due to the minimal risk of the data collection (i.e. breath test). No study-related medical intervention will be applied. The subjects involved in this research project will not be exposed to more than a minimal additional risk due to the study and will be medically treated as any subject not enrolled in the study.

2.3 Ethics Committee (EC) and Competent Authorities (CA), FOPH

Before the project will be conducted, the protocol and any substantial amendments, the study patient information and consent form as well as other relevant or requested material will be reviewed and approved by the responsible Cantonal Ethics Committee "Ethikkommission Nordwest – und Zentralschweiz (EKNZ)". Reporting duties and allowed time frames (all changes in research activity and all unanticipated problems involving risks to humans; including the case of planned or premature project end and the final report) will be handled according to the Human Research Act (HRA). The regular end, premature end or interruption of the research project will be reported to the EKNZ within 90 days upon completion of the project.

2.4 Participant Information and Informed Consent

Parents/legal representatives of children, as well as patients of 14 years old and above, requiring TDM will be informed about the study by the responsible clinician. Parents/legal representatives and their child will then be asked about their potential interest in the study by an investigator in person or by phone. The study team will approach interested patients and their parents/legal representatives and explain the rationale, the set up, and the risks and burden of being enrolled in the study and will be available for further questions and clarifications. In case parents /legal representatives and the patient are willing to contribute to the study, the informed consent document will be signed by the parents/legal representatives and, if applicable, by the child himself/ herself. Signed forms will be kept as part of the study file and will be available for verification at any time. A copy of the informed consent will be handed out to the parents/legal representatives. Study participation is voluntary and parents/legal representatives/ children can recall the informed consent at any time. There will be no direct benefit for children taking part in the study. The patient and/or the parents/legal representatives

have the right to withdraw from study participation at any time, without disclosing any reason and without any impact on the further medical treatment.

2.5 Participant privacy and safety

This study will be conducted in accordance with all applicable privacy laws and the principle of participating children's right to dignity, privacy and health will be upheld. Confidentiality of the participating children will be maintained by assigning a study number, keeping identifiers separate from the data and storing data in a locked file and secure computer database. Scientific reports generated from the study will not contain information that would identify the participating children. After termination of the study, records will be archived for ten years and then destroyed.
All personal and medical information obtained for this study is confidential and disclosure to third parties other than those noted below is prohibited. Participating children's data will be identified by study abbreviation and subject ID number (e.g. BreathTDM_001).
Upon the permission of parents/legal representatives, medical information may be given to the personal physician of the child or other appropriate medical personnel responsible for her or his welfare.
Records and documents pertaining to the conduct of this study, including Case Report Forms (CRFs), consent forms and clinical notes will be retained for 10 years.
For data verification purposes, authorized representatives of the sponsor, a competent authority, or an ethics committee will be granted direct access to parts of the medical records relevant to the project, including participating children's medical history.

2.6 Early termination of project

This is a pilot study with a planned sample size of 150 participating children. The sponsor and any competent authority may terminate the project prematurely according to certain circumstances, e.g. insufficient patient recruitment. However, due to thorough planning and a small initial sample size, we do not expect a premature end. In any case, a premature end or interruption of this project will be reported to the EKNZ within 90 days.

2.7 Amendments, Changes

Significant changes to the project plan will be submitted to the EKNZ for approval. Important research plan modifications (e.g. changes to eligibility criteria, outcomes, analyses) will be communicated to relevant parties.

3. INTRODUCTION

3.1 Background

The concept of personalized medicine revolves around tailoring treatment and care to the individual patient and their specific disease. In this context, therapeutic drug monitoring (TDM) is defined as measuring concentrations of a drug at one or more time points in a biological matrix after a dose. The purpose of TDM is to individualize the dose to achieve maximum efficacy of a drug and at the same time minimize toxicity (1). Chemotherapy and anti-seizure drugs are among the treatments that require TDM for optimizing dosing regimens (2, 3).
Breath is known to contain biochemical information on the metabolism of an individual, whereby both endogenous metabolites and xenobiotics are exhaled. With suitable analytical methods, the detection of these kinds of compounds in trace concentrations in breath is feasible (4, 5). This opens an opportunity to monitor drugs in breath, thereby allowing for non-invasive TDM, which is especially important in pediatric patients.

3.2 Rationale for the research project

Our goal is to obtain evidence that common drugs, requiring TDM, can be detected in exhaled air and that breath concentrations correlate with plasma concentrations. We hope that showing this proof-of-principle will lead to opportunities that will allow guiding the dose of drugs with TDM, based on a non-invasive breath test.

3.3 Risk-Benefit Assessment

The risk for the patients is minimal as this study involves a simple and non-invasive breath test by SESI-MS. This technique has been used during the last five years by the University Hospital Zurich without any risk for the patients, and more recently it has been extended to pediatric patients by the Children´s Hospital in Zurich. The SESI source is a consumer device and commercially available in Switzerland.
Our ultimate goal is to further develop this simple breath test to minimize or even circumvent other more invasive techniques used nowadays to monitor TDM (i.e. frequent blood sampling). Breath tests would also allow for more frequent sampling, thus enabling a more precise adjustment of the dose.

4. OBJECTIVES, ENPOINTS/OUTCOMES AND OTHER STUDY VARIABLES

4.1 Objectives

The primary objective of this study is to confirm whether TDM of selected drugs can be accomplished by real-time breath analysis.

Hypothesis
We hypothesize that drugs with sufficient volatility cross the blood-air barrier in the lung and are eventually exhaled in breath. In the equilibrium, and under ideal conditions, the concentration in blood correlates with that in the gas-phase, the relationship being the so-called Henry's constant. The hypothesis that breath-based tests can be used to monitor pharmacokinetic curves of drugs is supported by our previous work (5). We showed how ranges of drugs, including the anti-seizure drug valproate, could be detected in mice breath shortly after injecting the drugs. Moreover, measured plasma concentrations correlated significantly with breath signal intensity. Independent studies in humans using similar breath analysis techniques also found a strong linear correlation (*i.e.* $r > 0.9$) between breath and blood levels of valproic acid (6), ethanol (7) and propofol (8).

4.2 Primary and secondary endpoint/outcome(s)

Primary endpoint:
1) Correlation between drug concentrations in plasma/serum as measured by gold-standard methods and breath concentrations as measured by SESI-MS.

Secondary endpoints:

2) Evaluation of response to medication and drug toxicity as a function of blood/breath drug concentrations.

4.3 Other study variables

Potential factors affecting the metabolism of drugs (e.g. liver complications) or other unforeseen factors will be investigated in an explanatory manner as part of the primary analysis.

5. PROJECT DESIGN

5.1 Type of research and general project design

This is a single center prospective study. Health-related personal data are collected. Written informed consent will be obtained from all participating children's legal representatives before enrolment and after being informed of nature, purpose, and potential risks, both verbally and in writing. This study will be conducted at the University of Basel Children's Hospital.

5.2 Procedures

5.2.1 Overview

For timing and schedule of all study-related procedures, please refer to "Schedule of Assessments" (Table 1). The overall study duration has to be extended and will be undefined until all participants per group could be included.

5.2.2 Study description

We will conduct an observational study comparing exhaled drugs concentrations and plasma concentrations during therapeutic treatment. This observational study will be implemented in a single center (i.e. UKBB). We will recruit patients requiring TDM of their medication. In these patients, TDM peak and/or trough blood concentration (i.e. 15-30 minutes prior to the next dosage) will be collected routinely per standard of care. We will perform a breath test just before a blood sample is drawn during routine clinical TDM. Please note that therefore such blood measurements are done as part of the standard clinical care and are not per se part of this study. Repeated breath sampling for each subject will be conducted in the case that they require blood-based TDM per standard care on multiple times. Occasionally, we will also collect the exhaled breath condensate (EBC) for later analysis. Further data analysis will be conducted to assess whether there exists a significant correlation between plasma and breath concentrations.

Day -30 to -1

Patients requiring TDM per standard care will be asked to participate in this study. A clinician will explain the study to patient and parents/ legal representatives. If the parents/legal representatives are interested in participating in this study, the patient and the parents/legal representatives will receive an information flyer on the study and will inform them that they will be contacted by the study team. The clinician will then inform the study team about the potential patient.

The study team will then contact the patient's parents/legal representatives and explain the study procedures either in person at the clinic or in a phone call. If the parents/legal representatives are still interested, inclusion and exclusion criteria will be checked. The parents/legal representatives will be given enough time to consider participation in the study. If the parents/legal representatives agree to participate in the study, time and location for a face-to-face meeting will be arranged. The informed consent form will be signed at that occasion.

Day 0

Real-time SESI-MS

The patient comes to UKBB for a routine TDM test whereby a blood specimen is drawn. Shortly before or after the standard blood test, the patient will exhale into a SESI-MS breath analysis platform (9, 10). SESI-MS allows for real-time breath-printing by detection of both volatile and non-volatile trace components in breath without any sample pre-treatment. The SESI mass spectrometer is situated in a separate, lockable room at UKBB. Access to the designated room is only permitted to members of the study staff. Participants will be asked to refrain from eating, drinking, chewing gum use or brushing their teeth at least 1 hour before the measurements will be performed. Room temperature and lighting will be set at the same level for all measurements. Participants will exhale through a disposable mouthpiece into a commercially available SESI source (FIT S.L., Spain). While performing full exhalations, the subjects will keep the pressure through the sampling line at a fixed value monitored by a digital manometer. The flow entering the instrument will be controlled with a mass flow controller, ensuring as a result comparable results. Breathprints will be collected in real-time recording at least two replicates. The whole procedure is absolutely non-invasive and is usually accomplished without any effort in around 10 min per subject. Repeated breath sampling for each subject will be conducted in the case that they require blood-based TDM per standard care on multiple times.

Off-line SESI-MS
In addition, we will develop an off-line collection method with gas-tight syringes or sampling bags. Breath of patients on ICU with a central venous or peripheral arterial line will be collected in gas-tight syringes or gas-sampling bags for subsequent SESI-MS analysis of the collected air. In these patients, frequent blood sampling is routinely done to estimate the pharmacokinetic AUC (area under the curve). For these patients, every time point for which blood analysis is required per standard care, we will at the same time collect a breath sample. This procedure is even less invasive as it does not require an active exhalation from the patient. This off-line method will be implemented for the 0-5 years old subgroup in any case and in patients not capable of coming to the SESI-MS room (e.g. certain oncology patients). Repeated breath sampling for each subject will be conducted in the case that they require blood-based TDM per standard care on multiple times.

EBC Sampling
In parallel, we will also collect and store the exhaled breath from selected patients in liquefied form (exhaled breath condensate; EBC) for later analysis. To collect the breath samples selected patients will be asked to exhaled into a cold trap for roughly 10 minutes. This procedure has been already used by our group in the past (11) and is also completely non-invasive. EBC samples will be stored in -80 °C till further analysis. We plan to use these samples for confirming metabolite identification via running classical liquid chromatography-tandem mass spectrometry (LC-MS/MS) analyses.

Questionnaire
The patients will further respond to the ISAAC (International Study of Asthma and Allergies in Childhood) questionnaire, a standardized and validated questionnaire to assess respiratory and allergic outcomes (12). In the case in which repeated breath sampling for one subject is conducted, no additional questionnaire will be required. In the case in which the questionnaire cannot be accomplished, the blood and breath analyses data could still be used for the study.

Day 1-30
If the questionnaire cannot be completed on the day of the breath test, the patient will be asked to do so within 30 days.

Limitations
The main limitation of this study is that very little is known regarding whether drugs requiring TDM are capable of crossing the blood-air barrier in the lung. It is therefore difficult to anticipate whether the drugs will ultimately be detectable in exhaled breath with a suitable and sensitive analytical platform. This pilot study will shed light on this very issue.

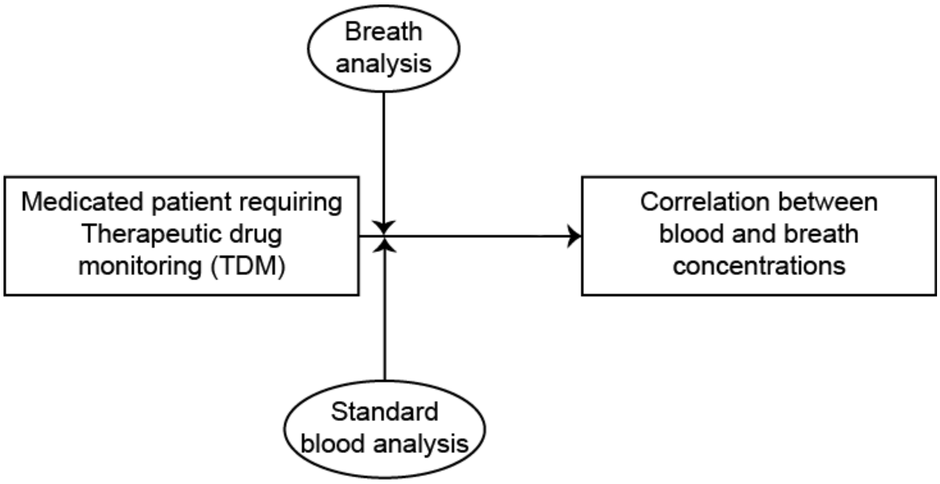

Figure 1. Schematic of the study flow. We will start the project with the sub-group of 13-22 yrs, we will continue in the next phase with 6-12 yrs and at the end of the study with 0-5 yrs, for which an off-line breath sampling methodology will be employed.

5.3 Recruitment and Screening

Patients from the outpatient clinic, ICU or regular wards of the UKBB will be asked to participate by a physician who is involved in the medical care of the patient. Potential participants will be identified for study enrolment according to the eligibility and ineligibility criteria outlined below. Essentially, these will be patients requiring plasma-based TDM per standard or care. These subjects (if applicable) and their parents will be given the patient information, and will be invited to meet a member of the trial team if they are interested in taking part. The study will be explained by a physician who is involved in the medical care of the patient. The study team then contacts the parents/legal representatives. If necessary, written information material (study flyer, information for patients) will be sent to the family. The study team will then inform them about the procedures of the study, assesses the inclusion and exclusion criteria according to a standardized screening sheet and asks for their informed consent. After informed consent has been obtained, inclusion and exclusion criteria will be re-assessed. Patients will then perform the exhaled breath analysis. Of the three age subgroups, we will start with those in the age range of 13-22, then 6-12 and finally 0-5 years old. For the latter group, only off-line breath collections methods will be used.

5.4 Methods of minimising bias

In order to minimize any source of bias, the researcher measuring the breath levels will not know the actual nor the expected blood levels of the drug investigated at the time of the breath test.

6. PROJECT POPULATION

The project population consists of 150 children of 0-22 yrs of age, divided into three age groups (0-5 yrs, 6-12 yrs, 13-22 yrs)

6.1 Inclusion criteria

1. Age 0 to 22 yrs old, divided into three age groups (0-5, 6-12 and 13-22 yrs old).

2. Treatment with anti-seizure medication(s) and/or chemotherapeutic agents and/or antibiotics that need therapeutic drug monitoring (TDM).

3. Patients undergoing therapeutic treatment with at least one of the following drugs:

- Chemotherapy:
  1. Methotrexate
  2. Busulfan
  3. Sirolimus

- Anti-convulsants:
  4. Valproate
  5. Oxcarbazepine
  6. Levetiracetam
  7. Sultiame
  8. Ethosuximide
  9. Lamotrigine
  10. Phenobarbital
  11. Perampanel
  12. Topiramate
  13. Carbamazepine
  14. Vigabatrine

- Antibiotics:
  15. Amikacin

4. Patients requiring blood/plasma-based TDM of the above listed drugs.

6.2 Exclusion criteria

1. Patients not capable of providing an exhalation into the mass spectrometer nor their breath can be sampled with off-line methods.
2. Inability of the care givers to understand the study.

6.3 Criteria for withdrawal / discontinuation of participants

- Patients' or parents'/legal representatives' withdrawal of informed consent.
- Patient or parents/legal representatives not willing to include their data into analysis after study completion.
- Technical issues: failure of the breath test.

7. PROJECT ASSESSMENTS

7.1 Project flow chart(s) / table of procedures and assessments

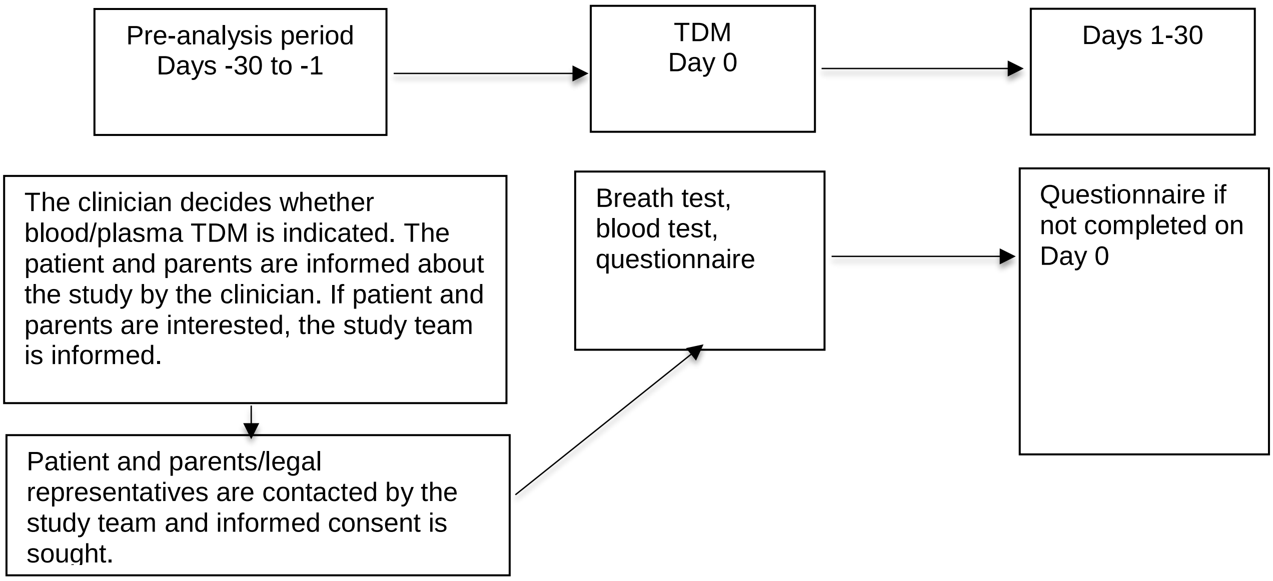

7.2 Assessments of primary endpoint/outcome

Blood-based drug concentrations
TDM peaks/trough concentrations will be measured following using reference methods, such as liquid chromatography combined with tandem mass spectrometry (LC-MS/MS). Established protocols for the drugs listed above are in place at UKBB/University Hospital Basel.

Breath-based drug concentration
We will monitor the ion intensity for the drugs of interest just before the blood sampling. Gas-phase ion concentrations will be assessed by calibrating the SESI-MS platform with known amounts of the analytes as instructed in our previous quantitative studies (13). If the absolute quantification procedure could not be accomplished, relative concentrations based on signal intensity will be used.

Finally, the assessment of the primary outcome will be based on the correlation between plasma and breath concentrations of the drugs investigated.

7.3 Assessment of secondary endpoints/outcomes

Toxicity/efficacy towards medication
Assessment of the secondary end-point will be the determination of drug toxicity/adverse reactions, and efficacy at the time of the breath/blood measurement will be based on clinical evidence.

7.4 Assessment of other study variables

Not applicable

7.5 Assessment of safety and reporting

7.5.1 Definition of Serious Events (SEs)

A serious event is any unfavorable event for which a causal relationship to sampling of biological material or the collection of health related personal data cannot be ruled out, and which:

- requires hospitalization or prolongation of an inpatients' hospitalization,
- results in persistent or significant disability or incapacity, or

- is life-threatening or results in death,

If a serious event occurs the research project will be set on hold.

7.5.2 Assessment and Documentation of SEs

Since the patients are receiving the drug anyways and their blood is analyzed per standard care, it is unlikely that they will experience any SE due to the study procedures (i.e. breath test). In other words, any SE will most likely be unrelated to the breath test. Nonetheless, if there is any SE, this study will be put on hold or stopped. All SEs will be documented in the participants´ file and on the SE report form.

7.5.3 Reporting of SEs, Safety and Protective Measures

The project leader shall report any occurring SE to the responsible EC within 7 days (and to the FOPH in case of involved radioactive sources). He shall also submit a report which evaluates the relationship between the event reported and the methods of collecting health related personal data or sampling of biological material within that project, furthermore proposals how to proceed with the project.
The project leader shall notify the EC within 7 days of any immediate other safety and protective measures, which have to be taken during the conduct of the research project. In addition, the project leader shall explain the circumstances, which necessitated the safety and protective measures.

8. STATISTICAL METHODOLOGY

8.1 Determination of Sample Size

8.1.1 Summary of the literature

Article	Year	Correlation coefficient between breath and blood concentrations	Drug
Miekisch *et al.* (8)	2008	r = 0.92 (n=16)	Propofol
Gamez *et al.* (6)	2011	r = 0.94 (n=7)	Valproic acid
Li *et al.* (5)	2015	r = 0.97 (n=4)	Ketamine

8.1.2 Hypothesis

- Gold standard: Plasma/blood concentrations for TDM.
- This is an exploratory study seeking to show “proof the principle” of breath TDM feasibility. We hypothesize that concentrations of drugs in exhaled air correlate linearly with concentrations in plasma/blood. This hypothesis is supported by long-lasting studies showing a clear association between breath and blood alcohol concentrations. For example, recent studies with modern analytical instrumentation showed a high correlation coefficient between breath and blood alcohol concentration levels (r = 0.983; n = 242; (7)). The same applies for common drugs such as for example propofol (r = 0.92; n = 16; (8)). In addition, in a previous preliminary study from our group, we injected selected drugs to mice and monitored their breath concentration (5). This study with mice showed significant ($p < 0.05$) linear correlations between breath signal intensity and plasma concentrations. The Person’s correlation coefficient we found was r = 0.97, which is in line with another breath analysis study in humans (6) using a similar technique to SESI-MS, for which they found an r = 0.94 for valproic acid. All these previous studies strongly indicate that correlation coefficients as high as r = 0.9 are to be expected between breath and blood concentrations of administered drugs.
- The sample size in this study is determined based on Monte Carlo simulations generating 10,000 samples of size 3 to 25 to test the correlation between the two variables: breath and blood concentrations. Based, on the previous breath/plasma studies mentioned above (5-8), we anticipate high correlations between blood and breath concentrations (*i.e.* r > 0.9). For a given sample size, we generated Monte Carlo simulations to determine an approximate cutoff value for a test of the correlation. We then generated samples under the alternative hypothesis, and estimated the power of the test.

Null hypothesis: Breath and plasma concentrations are uncorrelated.
Alternative hypothesis: Breath and plasma concentrations are correlated with r at least as high as 0.7.

8.1.3 Sample size calculation

Test for linear correlation between two variables by Monte Carlo simulations.

- Significance level = 5%
- Three different scenarios of power: 80, 85 and 90%
- Three different scenarios of correlation: r = 0.7, 0.8 and 0.9
- 95% confidence intervals for correlation computed using Clopper-Pearson method (14).

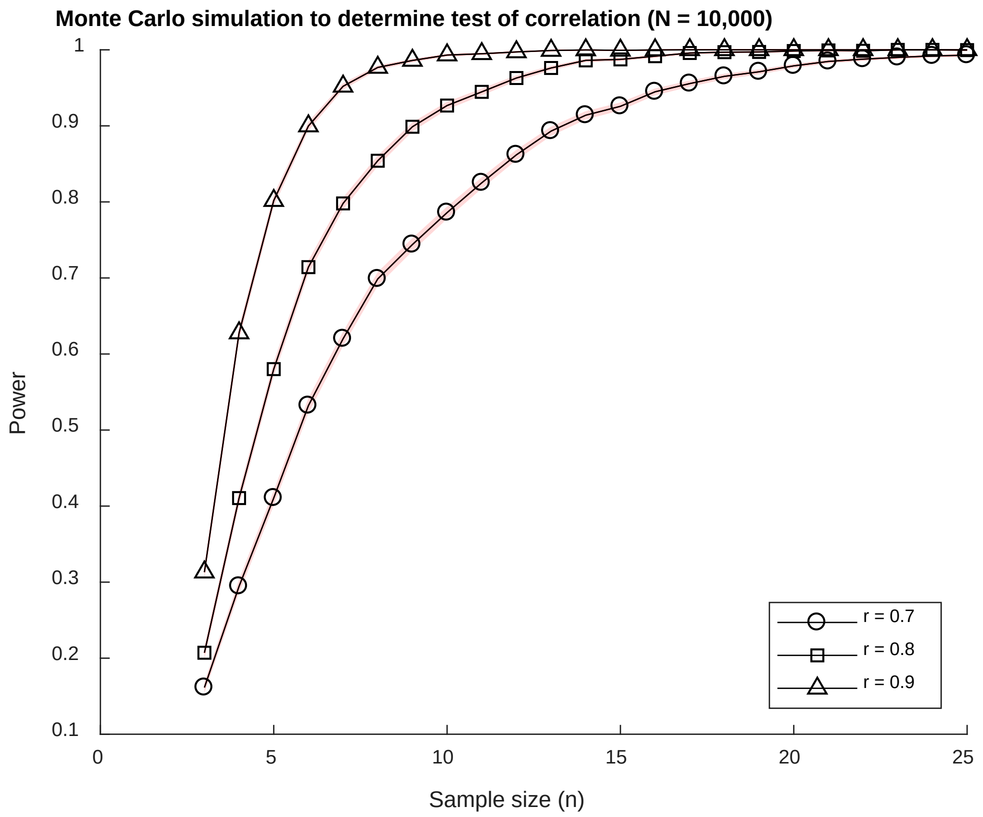

Figure 2. Power vs sample size for correlation test.

Correlation coefficient (r)	Scenario 1 = 80% power	Scenario 2 = 85% power	Scenario 3 = 90% power
0.7	10	11	13
0.8	7	8	9
0.9	5	5	6

Table 1. Sample size required for the three correlation coefficient considered here to achieve a power of 80, 85 and 90%

Correlation coefficient (r)	Power (n = 10)	95% CI	
0.7	0.800	0.792	0.808
0.8	0.926	0.921	0.931
0.9	0.994	0.992	0.995

Table 2. Expected power and 95% confidence interval for the three correlation coefficient considered here for 10 samples.

8.1.4 Drop-out considerations

We envisage the following reasons for patient not to complete the breath test:

1. Technical failure of the SESI-MS analytical platform
2. Patient cannot provide a full exhalation into the mass spectrometer

8.1.5 Final sample size for the primary endpoint

Assuming a significance level of 5%, with 90% power, the goal of detecting a correlation of at least r = 0.8 and accounting for a possible drop-out of 10%, we recommend a sample size of **10** patients per drug investigated (except for Levetiracetam and Valproate). Hence, as the total number of drugs in 15, we recommend a total of **150** patients to cover all the drugs.

8.2 Data processing

Raw mass spectra will be post-processed to extract the peak intensity for the selected studied drugs. Non-normal continuous data will be suitably transformed (e.g. Box-Cox) to achieve at least approximate normality.

8.3 Planned analysis

To test the hypothesis that there is a linear correlation between blood and breath drug concentrations, we will compute Pearson's correlation coefficient for blood- vs. breath-based drug concentrations. We will finally compute p-values for testing the hypothesis that there is no relationship between the observed phenomena (null hypothesis). The preprocessing of the raw mass spectra and the statistical analysis will be implemented using MATLAB .

8.3.1 Datasets to be analysed

Each of the 15 drugs studied will be analyzed as independent data sets.

8.3.2 Handling of missing data

This is an observational study looking at physiological outcomes, thus it will be analyzed on a per-protocol basis.

8.3.3 Ancillary analysis

Not applicable

8.3.4 Deviations from the original statistical plan

If substantial deviations from the planned analysis, as outlined in this document, are required for whatever reason, the protocol will be amended accordingly. Any extreme values will be investigated to determine if the patient differs significantly in any way from other patients. All deviations of the analysis from the protocol or from the detailed analysis plan will be listed and justified in a separate section of the final statistical report. The full analysis set and complete cases may nevertheless be used for additional and exploratory analyses.

9. DATA AND QUALITY MANAGEMENT

9.1 Data handling and record keeping / archiving

A designated research fellow will be responsible for data collection and data management. Data are registered in a case report form (CRF). Paper documents and informed consent forms will be stored in a lock-secured cupboard in a dedicated research office at the UKBB. Only study investigators have access to these documents. Authorized staff of the responsible ethics committee can request access to these data for monitoring and auditing purposes. Electronic data will be stored in a password-secured coded spreadsheet file. The file will be stored within the UKBB intranet server. It can only be accessed by authorized users from registered computers at the UKBB.

All personal and medical information obtained for this study is confidential and disclosure to third parties other than those noted below is prohibited. Confidentiality of the subjects will be maintained by assigning subjects a study number (ID), keeping identifiers separate from the data and storing data in a locked file and secure computer database in line with Swiss legal requirements. Patient identifier will be assigned with a consecutive number after the project acronym (e.g. BreathTDM_001).
Scientific reports generated from the study will not contain information that would identify the participating children. After termination of the study records and documents pertaining to the conduct of this study, including CRFs, consent forms, laboratory test results and clinical notes will be archived for ten years and then destroyed. The Division of Paediatric Pulmonology will handle administration of the study database. The data entry will be performed by the study team, consisting of investigators and study nurses. Study researchers will only be given access to database exports and will not have direct system access. Only de-identified database exports will be made accessible to the project collaborators.

9.2 Confidentiality, Data Protection

Data generation, transmission, storage and analysis of health related personal data within this project will follow strictly the current Swiss legal requirements for data protection and will be performed according to the Ordinance HRO Art. 5.
For data verification purposes, monitoring, audits or inspections, authorised representatives of the sponsor, a competent authority, or an ethics committee will be granted direct access to parts of the medical records relevant to the project, including participating children's medical history.

9.3 Coding

Health related personal data captured during this project from participating children are strictly confidential and disclosure to third parties is prohibited; coding will safeguard participating children' confidentiality. Project data will be handled with uttermost discretion and only be accessible to authorised personnel. Upon the permission of parents/legal representatives, medical information may be given to his or her personal physician or other appropriate medical personnel responsible for her or his welfare. The code may only be broken if it is necessary to avert an immediate risk to the health of the person concerned or to guarantee the rights of the person (e.g. in revoking the consent) or a legal basis exists for breaking the code.

9.4 Archiving and Destruction

Records and documents pertaining the conduct of this study, including CRFs, consent forms, patient diaries and clinical notes will be retained for 10 years.

10. PUBLICATION AND DISSEMINATION POLICY

10.1 Publication of results

We plan to publish the results of this study in a peer reviewed journal. We also plan to present the results of this study at clinical conferences.
Authorship credit is based on 1) substantial contributions to conception and design, acquisition of data, or analysis and interpretation of data; 2) drafting the article or revising it critically for important intellectual content; and 3) final approval of the version to be published.
Each author should have participated sufficiently in the work to take public responsibility for appropriate portions of the content.

10.2 Data sharing

As there are no associated partners for this project, no data sharing will be required.

11. FUNDING AND SUPPORT

This project will be supported by the Fondation Botnar via the Professorship of the principal investigator. We declare no conflict of interest (independence, intellectual, financial, proprietary).

12. INSURANCE

Studies in category A are exempt from the requirement of an insurance.

13. REFERENCES

1. Dasgupta A. Chapter 1 - Introduction to Therapeutic Drug Monitoring: Frequently and Less Frequently Monitored Drugs. Ther Drug Monit. Boston: Academic Press; 2012. p. 1-29.
2. Luke M. Chapter 12 - Therapeutic Drug Monitoring of Classical and Newer Anticonvulsants A2 - Dasgupta, Amitava. Ther Drug Monit. Boston: Academic Press; 2012. p. 243-267.
3. Milone MC. Chapter 14 - Therapeutic Drug Monitoring of Selected Anticancer Drugs: Pharmacogenomics Issues A2 - Dasgupta, Amitava. Ther Drug Monit. Boston: Academic Press; 2012. p. 291-321.
4. Martinez-Lozano Sinues P, Zenobi R, Kohler M. Analysis of the exhalome: A diagnostic tool of the future. *Chest* 2013; 144: 746-749.
5. Li X, Martinez-Lozano Sinues P, Dallmann R, Bregy L, Hollmen M, Proulx S, Brown SA, Detmar M, Kohler M, Zenobi R. Drug Pharmacokinetics Determined by Real-Time Analysis of Mouse Breath. *Angew Chem Int Ed* 2015; 54: 7815-7818.
6. Gamez G, Zhu L, Disko A, Chen H, Azov V, Chingin K, Kramer G, Zenobi R. Real-time, in vivo monitoring and pharmacokinetics of valproic acid via a novel biomarker in exhaled breath. *Chem Commun* 2011; 47: 4884-4886.
7. Jaffe DH, Siman-Tov M, Gopher A, Peleg K. Variability in the Blood/Breath Alcohol Ratio and Implications for Evidentiary Purposes. *J Forensic Sci* 2013; 58: 1233-1237.
8. Miekisch W, Fuchs P, Kamysek S, Neumann C, Schubert JK. Assessment of propofol concentrations in human breath and blood by means of HS-SPME-GC-MS. *Clin Chim Acta* 2008; 395: 32-37.
9. Martínez-Lozano P, Fernández de la Mora J. Electrospray ionization of volatiles in breath. *Int J Mass spectrom* 2007; 265: 68-72.
10. Martinez-Lozano P, Fernandez de la Mora J. Direct analysis of fatty acid vapors in breath by electrospray ionization and atmospheric pressure ionization-mass spectrometry. *Anal Chem* 2008; 80: 8210-8215.
11. Gaugg MT, Bruderer T, Nowak N, Eiffert L, Sinues PML, Kohler M, Zenobi R. Mass-Spectrometric Detection of Omega-Oxidation Products of Aliphatic Fatty Acids in Exhaled Breath. *Anal Chem* 2017; 89: 10329-10334.
12. Asher MI, Keil U, Anderson HR, Beasley R, Crane J, Martinez F, Mitchell EA, Pearce N, Sibbald B, Stewart AW, et al. International Study of Asthma and Allergies in Childhood (ISAAC): rationale and methods. *Eur Respir J* 1995; 8: 483-491.

13. Martínez-Lozano P, Rus J, Fernández de la Mora G, Hernández M, Fernández de la Mora J. Secondary Electrospray Ionization (SESI) of Ambient Vapors for Explosive Detection at Concentrations Below Parts Per Trillion. *J Am Soc Mass Spectrom* 2009; 20: 287-294.
14. Clopper CJ, Pearson ES. The use of confidence or fiducial limits illustrated in the case of the binomial. *Biometrika* 1934; 26: 404-413.

14. APPENDICES

- Study flyer
- Informed consent forms
- CTU report
