# Supplementary material for: Personalised therapeutic management of epileptic patients guided by pathway-driven breath metabolomics
Source: Commun Med (Lond). 2021 Aug 2;1:21. doi: 10.1038/s43856-021-00021-3 (PMC9053280; doi:10.1038/s43856-021-00021-3)
Supplement: Supplementary file 8 — Reporting Summary [file 43856_2021_21_MOESM8_ESM.pdf]

nature research

Corresponding author(s): Alexandre N. Datta, Pablo Sinues

Last updated by author(s): Jun 10, 2021

Reporting Summary

Nature Research wishes to improve the reproducibility of the work that we publish. This form provides structure for consistency and transparency in reporting. For further information on Nature Research policies, see our Editorial Policies and the Editorial Policy Checklist.

Statistics

For all statistical analyses, confirm that the following items are present in the figure legend, table legend, main text, or Methods section.

n/a	Confirmed	
☐	☒	The exact sample size (*n*) for each experimental group/condition, given as a discrete number and unit of measurement
☐	☒	A statement on whether measurements were taken from distinct samples or whether the same sample was measured repeatedly
☐	☒	The statistical test(s) used AND whether they are one- or two-sided<br>*Only common tests should be described solely by name; describe more complex techniques in the Methods section.*
☐	☒	A description of all covariates tested
☐	☒	A description of any assumptions or corrections, such as tests of normality and adjustment for multiple comparisons
☐	☒	A full description of the statistical parameters including central tendency (e.g. means) or other basic estimates (e.g. regression coefficient) AND variation (e.g. standard deviation) or associated estimates of uncertainty (e.g. confidence intervals)
☐	☒	For null hypothesis testing, the test statistic (e.g. *F*, *t*, *r*) with confidence intervals, effect sizes, degrees of freedom and *P* value noted<br>*Give P values as exact values whenever suitable.*
☒	☐	For Bayesian analysis, information on the choice of priors and Markov chain Monte Carlo settings
☒	☐	For hierarchical and complex designs, identification of the appropriate level for tests and full reporting of outcomes
☒	☐	Estimates of effect sizes (e.g. Cohen's *d*, Pearson's *r*), indicating how they were calculated

Our web collection on statistics for biologists contains articles on many of the points above.

Software and code

Policy information about availability of computer code

Data collection	Q Exactive Tune software (version 2.9) from Thermo Fisher Scientific was used to directly control mass spectrometer and record measurements.
Data analysis	ProteoWizard's msConvert (version 3.0.11233) was used to convert RAW files into mzXML file format and imported into MATLAB (version 2019b). The Combat function from sva (version 3.34.0) was used to remove known batch effects. MetaboAnalystR (version 2.0.4) was used to add biological insights into differentially abundant ions, by translating ions to metabolic pathways. Inhouse C# console app based on RawFileReader (version 5.0.0.38) an open source .Net assembly from Thermo Fisher Scientific was used to extract ions directly from RAW files. In house MATLAB scripts and functions were used for other analysis.

For manuscripts utilizing custom algorithms or software that are central to the research but not yet described in published literature, software must be made available to editors and reviewers. We strongly encourage code deposition in a community repository (e.g. GitHub). See the Nature Research guidelines for submitting code & software for further information.

Data

Policy information about availability of data

All manuscripts must include a data availability statement. This statement should provide the following information, where applicable:

- Accession codes, unique identifiers, or web links for publicly available datasets
- A list of figures that have associated raw data
- A description of any restrictions on data availability

All the data generated and analysed that support the findings in this study are within the article and its supplementary information files, and are available from the corresponding author upon reasonable request. Additionally, the RAW and mzXML files of the real-time breath measurements are available from the MetaboLights (https://www.ebi.ac.uk/metabolights) repository (accession number MTBLS2400).

Field-specific reporting

Please select the one below that is the best fit for your research. If you are not sure, read the appropriate sections before making your selection.

☒ Life sciences ☐ Behavioural & social sciences ☐ Ecological, evolutionary & environmental sciences

For a reference copy of the document with all sections, see nature.com/documents/nr-reporting-summary-flat.pdf

Life sciences study design

All studies must disclose on these points even when the disclosure is negative.

Sample size	Sample size determination was done prior to study, during ethical approval process. Monte Carlo simulations were used to estimate sample sizes under different scenarios of correlation between breath and blood drug levels. We estimated that with 10 measurements our predictions will have power between 0.79 - 0.99, if the correlation in breath and blood levels ranges between 0.7 - 0.9 (for more details see Supplementary Fig. 3).
Data exclusions	Data from failed breath measurements were excluded from further analyses. The exclusion criteria were pre-established and the reasons for failed measurements could be categorised as i) patients suffered from severe neurological impairment, preventing them to understand the instructions of the exhalation manoeuvre, or that the side effects would not allow them to perform the breath test (hence they are unable), ii) some technical issue with instrument during patient visit, or iii) in rare cases clinical laboratory could not return blood concentration of (any) antiseizure medications (Supplementary Fig. 1).
Replication	Exact replication of measurements was not possible due to involvement of real-life patients. However, we tested our prediction model in a completely independent data set from two different hospitals.
Randomization	Study did not include experimental groups in a classical sense, so no randomization was done, each subject takes one or combination of antiseizure medications as prescribed by their neurologist.
Blinding	Study does not involve group allocation during data collection and hence no blinding was performed. However, in order to minimize any source of bias, researchers measuring the breath levels did not know the actual or the expected blood levels of the drug investigated at the time of the breath test, but they new the drugs.

Reporting for specific materials, systems and methods

We require information from authors about some types of materials, experimental systems and methods used in many studies. Here, indicate whether each material, system or method listed is relevant to your study. If you are not sure if a list item applies to your research, read the appropriate section before selecting a response.

Materials & experimental systems

n/a	Involved in the study	
☒	☐	Antibodies
☒	☐	Eukaryotic cell lines
☒	☐	Palaeontology and archaeology
☒	☐	Animals and other organisms
☐	☒	Human research participants
☒	☐	Clinical data
☒	☐	Dual use research of concern

Methods

n/a	Involved in the study	
☒	☐	ChIP-seq
☒	☐	Flow cytometry
☒	☐	MRI-based neuroimaging

Human research participants

Policy information about studies involving human research participants

Population characteristics	66 paediatric epileptic patients (mean ± SD age, 10.7 ± 3.9 years; 37 males and 29 females) from the University Children's Hospital Basel (UKBB) and 41 adult epileptic patients (mean ± SD age, 51.6 ± 17.1 years; 29 male and 12 females) from the University Hospital Zurich (USZ) under treatment with various antiseizure medications (ASMs) requiring therapeutic drug monitoring per standard care were enrolled in this study. All subjects were under steady-state of their ASMs at the time of measurements.
Recruitment	Parents/legal representatives of epileptic children, as well as epileptic patients of 14 years old and above, requiring therapeutic drug monitoring were informed about the study by their neurologist. All subjects and/or parents, whichever applicable, signed informed consent to participate in the study in presence of their neurologist.
Ethics oversight	This study was approved by the Ethics Committee of North-western and Central Switzerland (ID 2017-01537) and the Cantonal Ethics Committee Zurich (ID 2019-00030).

Note that full information on the approval of the study protocol must also be provided in the manuscript.
