# Supplementary material for: Personalised therapeutic management of epileptic patients guided by pathway-driven breath metabolomics
Source: Commun Med (Lond). 2021 Aug 2;1:21. doi: 10.1038/s43856-021-00021-3 (PMC9053280; doi:10.1038/s43856-021-00021-3)
Supplement: Supplementary file 10 — Description of Additional Supplementary Files [file 43856_2021_21_MOESM10_ESM.pdf]

Description of Additional Supplementary Files

File Name: Supplementary Data 1
Description: Information about participants (source data for Fig. 2)

File Name: Supplementary Data 2
Description: Different matrices shown as heatmap in Fig. 1 (source data for Fig. 1)

File Name: Supplementary Data 3
Description: Source data for Fig. 3

File Name: Supplementary Data 4
Description: Source data for Fig. 4

File Name: Supplementary Data 5
Description: Source data for Fig. 5

File Name: Supplementary Data 6
Description: Source data for Fig. 6
